# Retromer-targeted therapy for neurodegenerative diseases

**DOI:** 10.1186/s13024-026-00971-z

**Published:** 2026-09-10

**Authors:** Michele Persico, Sophronea Tuithung, Alejandra Lorenzo, Darynaisha Crawford, Simona Eleuteri, David K. Simon

**Affiliations:** 1 Department of Neurology, Beth Israel Deaconess Medical Center, Harvard Medical School, Boston, USA; 2 Department of Neurology, Emory University School of Medicine, Atlanta, USA

**Keywords:** Alzheimer’s disease, Parkinson’s disease, Amyotrophic lateral sclerosis, Retromer, Neurodegeneration, Therapy

## Abstract

Alzheimer’s disease (AD), Parkinson’s disease (PD), and Amyotrophic Lateral Sclerosis (ALS) are neurodegenerative diseases characterized by dysfunction of the endosomal-lysosomal system (ELS). Four shared neurodegenerative mechanisms across ALS, PD and AD are regulated by the ELS, namely proteostasis and related protein misfolding, mitochondrial function, neurotransmission and neuroinflammation. These mechanisms are interconnected, contributing to neurodegeneration in a “snowball” manner. The retromer, a multimeric, evolutionarily conserved protein complex involved in intracellular protein trafficking, is at the crossroad of these neurodegenerative processes. This narrative review focuses on exploring the retromer structure, function as a master regulator of the ELS, and how this impacts proteostasis, mitochondrial biogenesis and homeostasis, neurotransmission and neuroinflammation across neurodegenerative diseases. We explore how alterations in retromer function can play an important role in neurodegeneration and discuss the impact of genetic and pharmacological manipulations of VPS35, one of the main retromer subunits. In vitro and in vivo studies have identified that the retromer enhances the activity of protein degradation pathways via the ELS, namely macroautophagy, chaperone-mediated autophagy, and the ELS itself, with concomitant reduction in misfolded protein levels. Also, in some model systems, when the retromer role is enhanced or restored, mitochondrial function is rescued, dysfunctional neurotransmission is restored, and the damaging effects of neuroinflammation are dampened. Lastly, we highlight the role of a novel pharmacological class of agents that enhance retromer function as a strategy for potentially slowing the progression of these neurodegenerative diseases in in vivo models of PD and ALS. We discuss the challenges in targeting the retromer, current limitations and potential off-target effects of retromer enhancement. Overall, the retromer regulates shared mechanisms across neurodegenerative diseases and retromer enhancers could represent a novel disease-modifying strategy in AD, PD and ALS.

## Introduction

This review analyzes the retromer complex-mediated modulation of the endosomal-lysosomal system (ELS) in Alzheimer’s disease (AD), Parkinson’s disease (PD) and Amyotrophic Lateral Sclerosis (ALS) and explores how this affects their pathophysiology. As such, this review provides a description of the retromer complex structure and assembly process and AD, PD and ALS pathophysiology. This is followed by an analysis of the pathophysiology of the endosomal-lysosomal system (ELS) in health and neurodegenerative diseases, the retromer’s role in it and a detailed analysis of the ELS-mediated retromer role in regulating cellular proteostasis, mitochondrial function, neurotransmission and neuroinflammation in AD, PD and ALS. This review ends with a discussion of the potential role of the retromer as a therapeutic target and challenges of targeting the retromer with small molecules.

### Retromer complex structure

The retromer is an evolutionarily conserved multimeric complex originally identified in the budding yeast *Saccharomyces cerevisiae* by Seaman and colleagues [1]. In yeasts, the retromer is a stable heteropentamer composed of five distinct proteins encoded by the vacuole protein sorting (*VPS*) genes, i.e. *VPS35*, *VPS26*, *VPS29*, *VPS5*, and *VPS17* [2]. The heterotrimer Vps35/Vps26/Vps29 recruits the heterodimer Vps5/Vps17 sequentially [1]. The process of retromer-mediated vesicle formation at the endosome starts with the binding of Vps35 to the aromatic tail of transmembrane endosomal proteins such as Vps10 or Kex2p [1]. Next, Vps29 is recruited and activates Vps35, which clusters with the cargo protein. This is the signal that recruits the Vps5/Vps17 heterodimer [1]. Vps5 binds both Vps29 and Vps35 subunits through its unstructured N-terminal domain [3]. The final structure of the retromer assembled on the endosomal membrane has been characterized in the thermophilic ascomycete *Chaetomium thermophilum* via cryo-electron tomography [4]. Each one of two copies of the heterotrimer Vps35/Vps26/Vps29 sits on a membrane-bound Vps5 interface and forms a dimeric arch [5]. Vps26 forms the “foot” between each Vps35 “leg” and Vps5 [5].

**Fig. 1:**
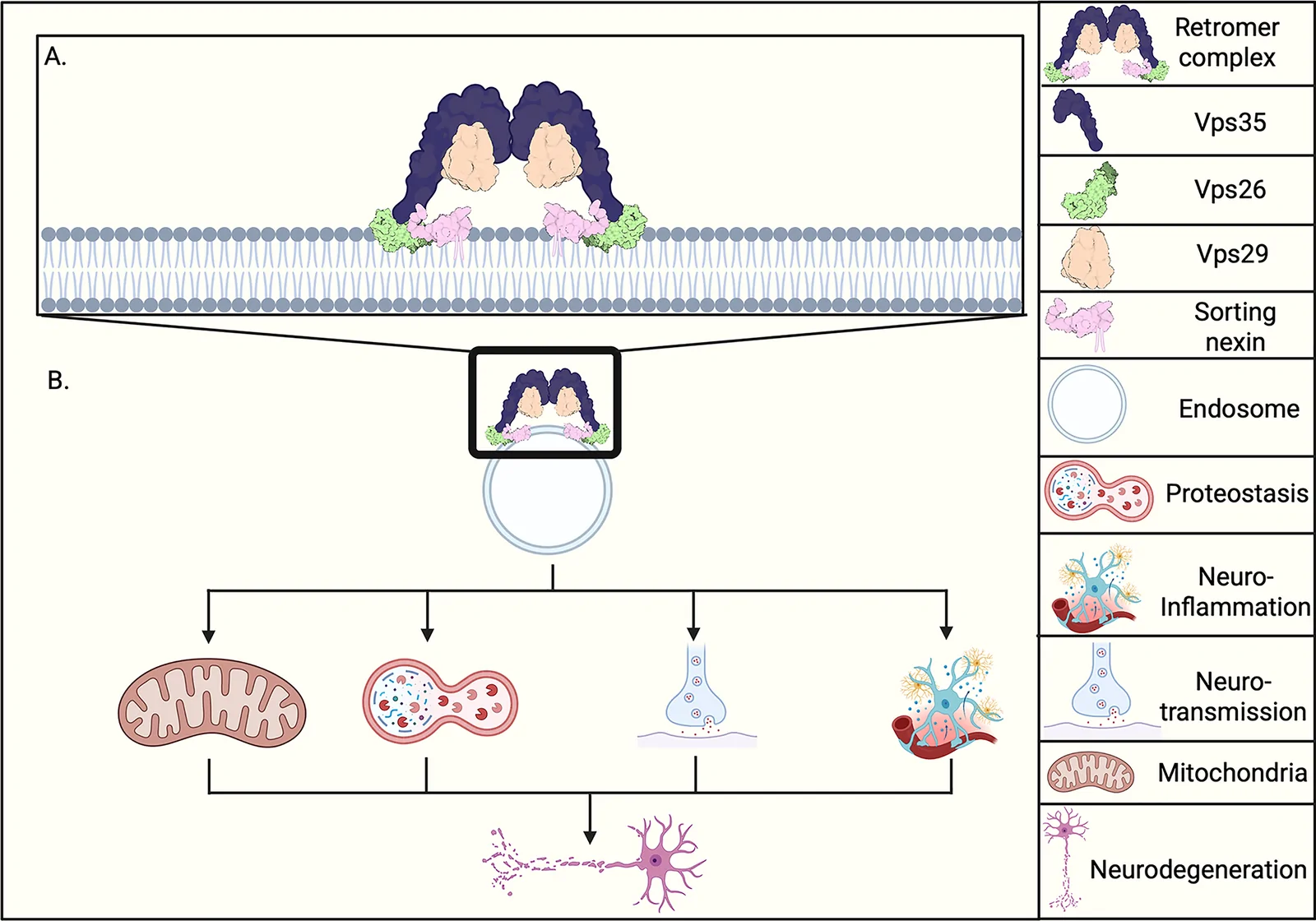
Retromer structure and its central role in neurodegeneration. (**A**) The mammalian retromer is composed of two copies of VPS35 and VPS29 dimers that form an arch on the VPS26 interface, with VPS26 forming the ‘‘foot’’ between each VPS35 ‘‘leg’’. With its α-solenoid structure, VPS35 binds VPS26 at its N-terminus and Vps29 at its C-terminus. Sorting nexins are the retromer subunits that bind to membrane phospholipids. (**B**) Via modulation of the endosomal-lysosomal system, the retromer regulates cellular proteostasis, mitochondrial function, neurotransmission and neuroinflammation, whose dysfunction is involved in the pathogenesis of neurodegenerative conditions

In mammals, the retromer has a 150-kD core heterotrimer composed of VPS26, VPS35, and VPS29 (formerly called the cargo-selection complex, CSC), which is responsible for binding transmembrane cargo proteins (Fig. 1) [6]. With its α-solenoid structure, VPS35 provides a flexible platform for binding VPS26 at its N-terminus and VPS29 at its C-terminus [7]. The stability of the heterotrimer depends on VPS35, and hence, the modulation of this protein is commonly used to study retromer function. In mammals, VPS26 exists in two isoforms, VPS26A and VPS26B, whereas VPS29 has three isoforms, VPS29A, VPS29B and VPS29C [8, 9]. The retromer binds a dimer of different sorting nexin (SNX) proteins, previously called the tubulation module, as SNXs drive tubule formation [6]. In addition, the SNX family of proteins serves as the scaffold for the retromer, which lacks a domain capable of binding membrane phospholipids (Fig. 1) [10]. The SNX family of proteins encompasses SNX with a Bin/Amphiphysin/Rvs (BAR) domain that binds phosphatidylinositol 3-phosphate (PI3P) and SNXs without a BAR domain (e.g. SNX3, SNX27) capable of interacting with PI3P through different domains (e.g. phox-homolog domain for SNX3) [10–12].

The retromer can bind the heterodimer composed of a combination of SNX1 or SNX2 with SNX5, SNX6, SNX3 and the metazoan-specific SNX27 [6]. SNX1/2 is a mammalian homolog of the yeast Vps5, whereas SNX5/6 is the homolog of the yeast Vps17 [13–15]. The heterodimeric complex composed of a combination of SNX1 or SNX2 with SNX5, SNX6 or SNX32 is also known as the endosomal sorting complex for promoting exit 1 (ESCPE-1), a complex distinct from the retromer with the ability to bind its own cargo [16]. ESCPE-1 is involved in the sorting of over sixty transmembrane protein cargoes in the ELS [16, 17]. ESCPE-1 cargoes are rescued from the early endosome (EE) and late endosome (LE) to the *trans*-Golgi network (TGN) and the plasma membrane [18, 19]. In a handover model, SNX27 associates with the retromer and cargo proteins captured by the SNX27-retromer are transferred to ESCPE-1 to promote endosome-to-plasma membrane recycling [20].

The nexin dimer is necessary but not sufficient to recruit the retromer on the endosomal membrane because of their weak interaction, so this requires a second key protein, the small guanosine triphosphatase (GTPase) RAB7a [21, 22]. The importance of RAB7a is confirmed by studies showing that the GTPase-activating protein TBC1D5 (TBC1 domain family member 5), by deactivating RAB7a, inhibits the retromer recruitment process [23, 24]. Interestingly, RAB7a localization is maintained by both the retromer and TBC1D5. In the absence of either TBC1D5, VPS35 or VPS29, RAB7a activity state and localization are no longer properly controlled [25]. The retromer is recruited to the endosomal surface by RAB7a and nexins and starts tubule formation by orchestrating the SNX proteins’ distribution on the endosomal membrane [21, 23, 26].

The process of endosomal tubulation and scission is facilitated by the Wiskott-Aldrich syndrome and SCAR homolog (WASH) complex [27]. This multimeric structure interacts with the retromer via the FAM21 (family with sequence similarity 21) tail of the protein WASH, with the binding site located on VPS35 and VPS29 [28, 29]. Interestingly, FAM21 also interacts with SNX27 and SNX1 [28, 30]. The SNX1 interaction happens via the protein RME8, and loss of this protein leads to impairment in the SNX1 association with the endosomal membrane and with the formation of branched endosomal tubules [30]. After the binding is achieved, additional actors take part in the polymerization of the actin network around the endosomal tubules, which contributes to endosomal tubulation and scission [31].

### Neurodegenerative diseases pathophysiology

The term neurodegenerative disease encompasses a wide range of pathologies impacting the peripheral and central nervous system, characterized by a progressive loss of neurons and altered function of glial cells. In this review, we focus on AD, PD, and ALS.

**Fig. 2:**
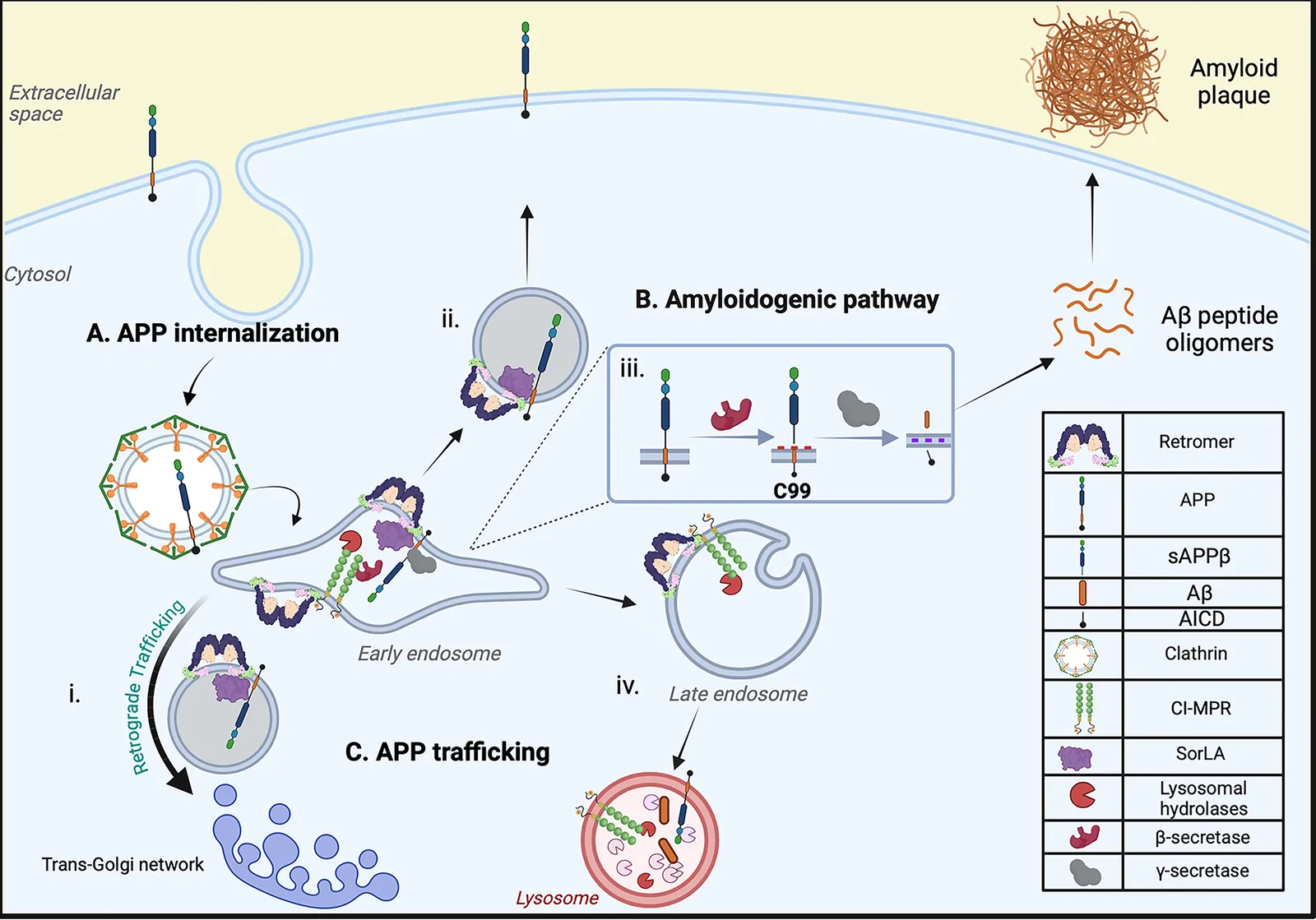
Retromer role in amyloid precursor protein (APP) processing in Alzheimer’s disease. (**A**) APP internalization: APP is internalized from the cell surface to the early endosomes via a clathrin-mediated mechanism. (**B**) Amyloidogenic pathway: APP undergoes a sequential cut first by β-secretases, releasing soluble APP β (sAPP β) and with the C99 residue remaining in the endosomal membrane. As the second step, γ-secretases cleave C99 in the Aβ fibrillary protein and the amyloid precursor intracellular domain (AICD). (**C**) APP trafficking: APP circulates between the trans-Golgi (TGN) network, early endosome and plasma membrane, or late endosome and lysosome. SORL1, via interaction with Vps26b, carries APP from endosomal compartments to the (i) TGN or (ii) plasma membrane, evading the intracellular amyloidogenic pathway; (iii) Endosomal defects associated with AD promote APP enzymatic processing by β-secretases, initiating the amyloidogenic pathway; (iv) Aβ and APP clearance: The retromer modulates the clearance of Aβ via the distribution of the cathepsin-carrying protein CI-MPR to late endosomes and lysosomes. Created in https://BioRender.com

*Alzheimer’s disease:* AD is the most common neurodegenerative disease in the United States [32] and the most common form of dementia worldwide in the older population [33]. AD pathogenesis starts with the internalization of the 770 amino acid transmembrane protein amyloid precursor protein (APP) from the cell surface to early endosomes. APP undergoes a sequential cut first by beta-site amyloid precursor protein cleaving enzyme 1 (BACE1, also known as β-secretases), releasing soluble APP β (sAPPβ) and with the C99 residue remaining in the endosomal membrane. As the second step, γ-secretases cleave C99 in the amyloid-β (Aβ) fibrillary protein and the amyloid precursor intracellular domain (AICD) [34] (Fig. 2). Aβ compromises synaptic signaling, triggers microglial activation via Aβ oligomers, and triggers the conversion of the microtubule-associated protein tau from a normal to a toxic state [35–37].

**Fig. 3:**
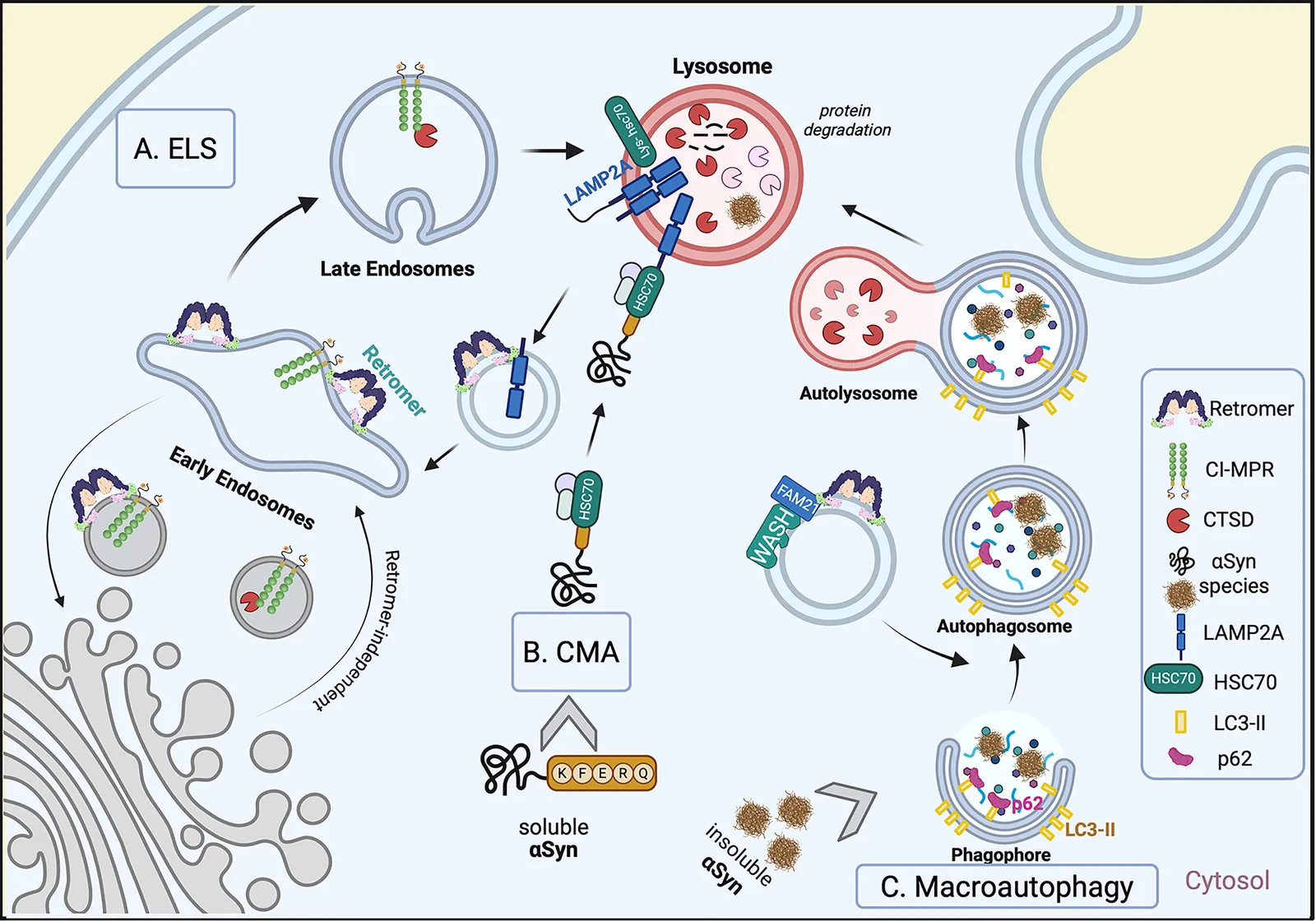
Retromer role in α-Synuclein degradation in Parkinson’s disease. (**A**) Endosomal-lysosomal system: Cathepsin D (CTSD) and cathepsin L (CTSL) are involved in the lysosomal degradation of α-Synuclein. Sortilin (not depicted) and CI-MPR are involved in the sorting of lysosomal hydrolases. The retromer regulates the sorting of CI-MPR between the trans-Golgi network, early, late endosomes, and lysosomes. The retromer controls the localization of RAB5 and RAB7, important mediators of the ELS. (**B**) Chaperone-mediated autophagy (CMA): Soluble α-Synuclein carries the KFERQ recognition motif recognized by the chaperone proteins Heat Shock Cognate 70 (HSC70), which sorts the substrate to Lysosomal-Associated Membrane Protein 2 A (LAMP2A). VPS35 regulates LAMP2A transport to and from the lysosome, and retromer enhancement increases levels of HSC70 and LAMP2A. (**C**) Macroautophagy: Insoluble α-Synuclein is degraded via macroautophagy. VPS35 governs macroautophagy by controlling the Wiskott-Aldrich syndrome protein and SCAR homolog (WASH) complex’s sorting and recycling by interacting directly with one of its subunits, the protein FAM21, and by modulating p62 and LC3-II levels. Created in https://BioRender.com

*Parkinson’s disease:* PD is the second most common neurodegenerative disorder, affecting 1% of people over 65 and 4.3% of those older than 85 [38]. Much of the motor symptoms, which are the basis of the clinical diagnosis of PD [38], are attributed to the preferential loss of dopaminergic (DA) neurons in the *substantia nigra* (SN), resulting in degeneration of the nigrostriatal tract and dopamine deficiency [39]. A convincing set of data from genetic, animal models, and biochemical studies supports the view that accumulation and aggregation of the protein α-synuclein (αS) in DA neurons may represent a key pathogenic event for PD (Fig. 3) [40]. αS is a protein usually present in the cytoplasm of neurons whose native conformation and related PD pathogenetic mechanisms are a matter of debate. Initial analysis revealed native αS as a 14 KDa unfolded monomer, with an imbalance between the αS rate of synthesis and clearance resulting in abnormal αS concentrations that might push the formation and accumulation of toxic oligomeric and fibrillar species [40]. Further studies highlighted the 58 kDa folded tetramer with an α-helical structure as the native αS form, and it has been proposed that destabilization of the tetramer precedes the αS misfolding and aggregation in toxic oligomeric and fibrillar species [41–43]. The αS fibrils release prefibrillar oligomers, which, due to solvent-exposed hydrophobic functional groups, are capable of permeabilizing neuronal membranes, leading to cellular insults [44].

**Fig. 4:**
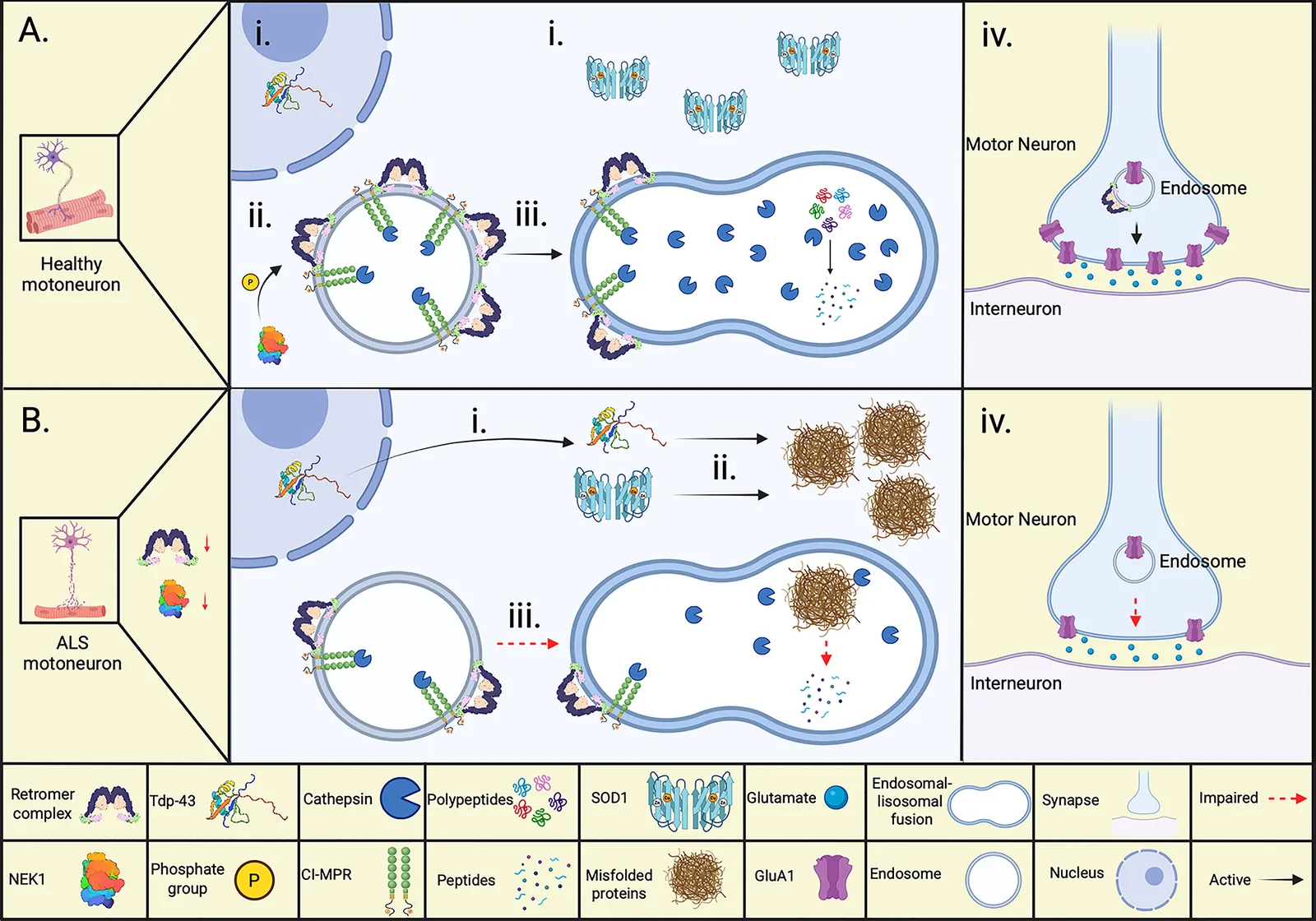
Retromer role in motor neurons. (**A**) Healthy motor neurons: (i) TAR DNA-binding protein 43 (TDP-43) and Superoxide Dismutase 1 (SOD1) maintain their physiological nuclear and cytosolic localization, respectively, without significant misfolding and aggregating; (ii) NIMA-related Kinase 1 (NEK1) regulates retromer-mediated endosomal trafficking by phosphorylating VPS26B; (iii) the retromer, via the interaction with Cation-Independent Mannose Phosphate Receptor (CI-MPR) which carries cathepsins, coordinates cathepsins transport to the lysosomes where they catabolize polypeptides in peptides; (iv) retromer facilitates the Glutamate Receptor Subunit A1 (GluA1, or GluR1) recycling to the synaptic membrane in the interface motor neuron-interneuron. (**B**) Amyotrophic Lateral Sclerosis motor neurons have decreased levels of retromer and NEK2: (i) TDP-43 mis-localizes to the cytosol, (ii) TDP-43 and SOD-1 misfold and form misfolded protein aggregates, (iii) a reduced amount of cathepsins is carried to the lysosomes, with consequent impaired enzymatic proteolysis, (iv) decreased levels of VPS35 limit GluA1 recycling on the synaptic membrane. Created in https://BioRender.com

*Amyotrophic Lateral Sclerosis:* ALS is a motor neuron disease characterized by the rapid progression of upper and lower motor neuron degeneration, resulting in progressive muscle weakness and eventually death [45]. The pathogenesis of ALS is only partially understood and partially based on pre-clinical data from familial forms of ALS. The superoxide dismutase 1 (SOD1) gene mutation is the second most common cause of hereditary forms of ALS, accounting for ~ 20% of familial ALS cases [46]. Mutant SOD1 protein tends to misfold, with subsequent formation of aggregates that lead to motoneuron injury and death (Fig. 4) [47]. Mutations in transactive response DNA-binding protein 43 (*TDP-43*), a gene for RNA-binding proteins, are also involved in familial ALS [48]. Mutated TDP-43 is found primarily in the cytoplasm instead of the nucleus, causing neurodegeneration (Fig. 4) [48]. TDP-43 mutations lead to misfolding and aggregation (Fig. 4) [49]. TDP-43 aggregates sequester the normal function of TDP-43, but these aggregates also actively cause splicing dysregulation, increases in DNA damage, transcriptome-wide changes, and disruption of oxidative stress response [50, 51]. Possible mechanisms described in ALS are oxidative stress, glutamate excitotoxicity, mitochondrial and proteasomal dysfunctions, altered synaptic function, disturbed axonal transport, and neuroinflammation [52, 53].

### Endosomal-lysosomal system dysfunction is the culprit of neurodegenerative diseases

AD, PD, ALS are neurodegenerative diseases characterized by progressive neuronal loss in selective central nervous system regions, namely the medial temporal lobe with hippocampal involvement, the lateral region of the parietal, temporal and frontal lobes in AD, the SN *pars compacta* in PD, the anterior horns of the spinal cord with lower motor neurons and the corticospinal tracts in ALS [54–57]. Despite the degeneration of different central nervous system (CNS) areas and neuronal types, similar pathophysiological mechanisms may ignite the cascade of cellular changes that lead to neuronal loss. A key pathophysiological mechanism that has been the focus of recent research on PD, AD and ALS includes endolysosomal trafficking and vesicle-mediated protein sorting [58–60].

The ELS is an intracellular membrane-enclosed system responsible for the trafficking of intracellular proteins, glycoproteins, and protein-bound lipid cargoes that play a critical role in maintaining cellular homeostasis [61]. It is composed of EE, LE, lysosomes and recycling endosomes [61]. Molecular sorting along the ELS is regulated by the RAB proteins, a family of small GTPases [62]. After internalization via clathrin-dependent mechanisms, among others, cargo proteins reach the EE under the modulation of RAB5, a marker of EE [63]. EE is a major hub, from which proteins can move to different destinations. The sorting to the plasma membrane or extracellular space via recycling endosomes is mediated by the retromer and involves RAB4 and RAB11 [64]. If the delivery from the EE is to the LE, there is a switch from RAB5 to RAB7 [65]. LEs fuse with lysosomes, where cargo proteins are degraded by lysosomal hydrolases [61]. This trafficking from EE to LE to lysosomes is mediated by the endosomal complex required for transport (or ESCRT) [66]. ESCRT recognizes ubiquitinated proteins, and the sorting process is ubiquitin-dependent [66]. Lastly, cargoes are trafficked from the EE to the TGN via the retromer. In fact, the term “retromer” refers to the first function discovered for this complex, namely the vesicular transport of transmembrane protein cargoes from the early endosome to the *trans*-Golgi network [1]. The retromer is also involved in endosomal protein trafficking from mitochondria to peroxisomes or lysosomes via mitochondrial-derived vesicles (MDVs). These vesicular carriers transport mitochondrial proteins and lipids to other intracellular organelles (Fig. 1) [67, 68]. Protein cargoes recycling from the endosomes to the plasma membrane are mediated by the handoff mechanisms from the retromer: SNX27 to the ESCP-1 complex, or by the interaction between the retriever complex and the COMMD/CCDC22/CCDC93 (CCC) complex [20, 69]. The retriever complex is a trimeric paralog of the retromer composed of the proteins VPS35 endosomal protein-sorting factor-like protein (VPS35L), VPS26C and VPS29 [69]. The CCC complex comprises the coiled-coil domain-containing (CCDC)22, CCDC93 and copper metabolism domain-containing (COMMD) proteins [69]. The retriever complex associated with SNX17 and couples to CCC and WASH complex to promote cell surface recycling [70].

Aging affects the ELS functionality. Age-related oxidative stress increases the amount of oxidized proteins within lysosomes, which impairs lysosomal activity by compromising the functionality of membrane organelles and leading to intralysosomal protein overload [71]. Additionally, endolysosomes increase in size and number due to acidification defects and aberrantly accumulate near synapses in aged neurons and brains [72]. Aging is also the primary risk factor for most neurodegenerative diseases [73]. Hence, it is unsurprising that an aged and dysfunctional ELS remains central in the processes of neurodegeneration. In AD postmortem brains, a cytopathologic hallmark of the disease is abnormal enlargement of early endosomes [74, 75]. This finding can be related to the dysfunction of the endosomal system, with an amyloid-independent traffic jam resulting in enlarged and dysfunctional endosomes, which subsequently mediates amyloid, tau, synaptic and microglia pathology [74–76]. In autopsy of sporadic PD brains, early endosomes maturation and trafficking are disrupted, lysosomes are changed in shape and size with electron microscopy highlighting swollen and fragmented lysosomes [77–79]. Mutations in several genes encoding ELS proteins, including VPS35, cause familial forms of PD. For example, glucocebrebrosidase (*GBA*) gene mutation leads to early-onset PD. *GBA* encodes for a lysosomal enzyme and its mutation leads to enlarged lysosomes, with increased electron-dense material, suggesting that their contents are not cleared appropriately [80]. As such, different authors argue that the ELS dysfunction drives αS pathology [81, 82]. Aberrant and enlarged endosomes are also observed in sporadic ALS tissues [83]. The classic ALS mouse model, the *wobbler* mouse, has a partial loss-of-function mutation in the *Vps54* gene, a component of the Golgi-associated retrograde protein (GARP) complex, which recycles cargoes from the LE to the TGN [84, 85]. Lastly, in ALS pre-clinical models, TDP-43 accumulation was found to be a downstream event secondary to the disruption of the endolysosomal pathway [86].

A dysfunctional ELS is associated with four mechanisms known to trigger neurobiological degenerative processes: (i) proteostasis with protein misfolding and aggregation, (ii) dysfunction of mitochondria biogenesis and homeostasis, (iii) neurotransmission and (iv) neuroinflammation (Fig. 1) [58–60]. These mechanisms have been identified as potentially relevant in ALS, PD and AD in a 2025 large-scale high-throughput proteomic study led by the Global Neurodegeneration Proteomics Consortium, highlighting converging biological pathways across neurodegenerative diseases centered around the ELS [87]. As such, a dysfunctional ELS represents an initial driving force upstream of these shared mechanisms. As a subsequent step, these initial dysfunctional pathogenic processes affect one another and themselves in a “snowball” effect, leading to neurodegeneration.

### The retromer is at the crossroads of neurodegenerative diseases

In this context, an emerging role in neurodegeneration has been identified for the retromer complex. The retromer complex plays a role in neuron development and survival, as demonstrated by a mouse model in which pan-neuronal deletion of VPS35 leads to early post-natal lethality and by another mouse model in which homozygous deletion of *VPS35* leads to early embryonic lethality [88, 89]. Correlative analysis between RNA-Seq and the whole cell proteome from *VPS35* knock-out (KO) cell lines has revealed disease signatures typical of PD, AD and ALS, among others [90]. To follow, we discuss the genetic data linking the retromer to AD, PD, and ALS.

*AD, retromer and genetics: *The retromer protein VPS35 is deficient in vulnerable regions in AD brains [91], and a rare *de novo VPS35* variant with missense mutation Leu625Pro has been identified in sporadic early-onset AD [92], as have variants in a range of other retromer-related genes [93]. The retromer subunit VPS26B was found to be enriched in the trans-entorhinal cortex of human brains and deficient in AD [94].

Amyloid precursor-like protein 2 (APLP2) and tau were among the most significantly enriched proteins in the cerebrospinal fluid (CSF) of *Vps35* KO mice [95]. Wen et al. have confirmed that mice with a hemizygous deletion of *Vps35* or *Vps26* heterozygote KO develop hippocampal-dependent memory and synaptic dysfunction associated with endogenous Aβ peptide accumulation [89, 96] due to an increase in BACE1 activity [89]. These data suggest a retromer loss-of-function as a pathogenetic event in AD and increasing retromer levels as a potential therapeutic strategy for countering AD pathology.

*PD, retromer and genetics: VPS35* is among the genes related to familial forms of PD. The most common *VPS35* mutation, the missense mutation Asp620Asn (D620N), is responsible for an autosomal dominant form of PD that is similar to its idiopathic form [97, 98]. Whether this mutation leads to a VPS35 loss of function, gain of function or both is still subject to debate. Alessi et al. demonstrated that VPS35 controls the Leucine-rich repeat kinase 2 (LRRK2) activity and that the *VPS35*^*D620N*^ mutation is associated with hyperactivation of the LRRK2 kinase and subsequent hyperphosphorylation of RAB8A, RAB10, and RAB12 [99]. Also, *VPS35*^*D620N*^ knock-in (KI) mice have been shown to have abnormal tau hyperphosphorylation, tau-positive somatodendritic pathology throughout the brain, and progressive degeneration of nigrostriatal dopaminergic neurons, suggesting another mechanism through which *VPS35*^*D620N*^ can lead to toxicity [100]. On the other end of the spectrum, the *VPS35*^*D620N*^ mutation impairs synaptic function, disrupts retromer cargo protein sorting, and impairs macroautophagy, chaperone-mediated autophagy, and the ELS, and alters mitochondrial homeostasis, highlighting also a potential loss-of-function role. These potential loss-of-function mechanisms will be addressed in the next paragraphs.

*ALS, retromer and genetics: *In ALS patients, different studies demonstrated a reduction in VPS35, VPS29, VPS26A levels [101]. This evidence was also confirmed in transgenic mice expressing the mutant SOD1 G93A [102]. Similarly, Muzio et al. demonstrate a significant reduction in the retromer subunits VPS35 and VPS26 in IPSC-derived motor neurons (MNs) from ALS patients, in the ventral horn MNs from ALS post-mortem explants, and in MNs from an ALS mouse model (SOD1^G93A^) [103]. This evidence is further strengthened by evidence of spinal cord motor neuron degeneration in conditional KO mice with selective deletion of *Vps35* in neurons [88]. The decrease in retromer subunit levels in the ALS mouse model is prodromal to MN degeneration, raising the possibility that the retromer complex could represent a trigger for neurodegeneration [103].

### The retromer is a master regulator of the endosomal-lysosomal system

**Fig. 5:**
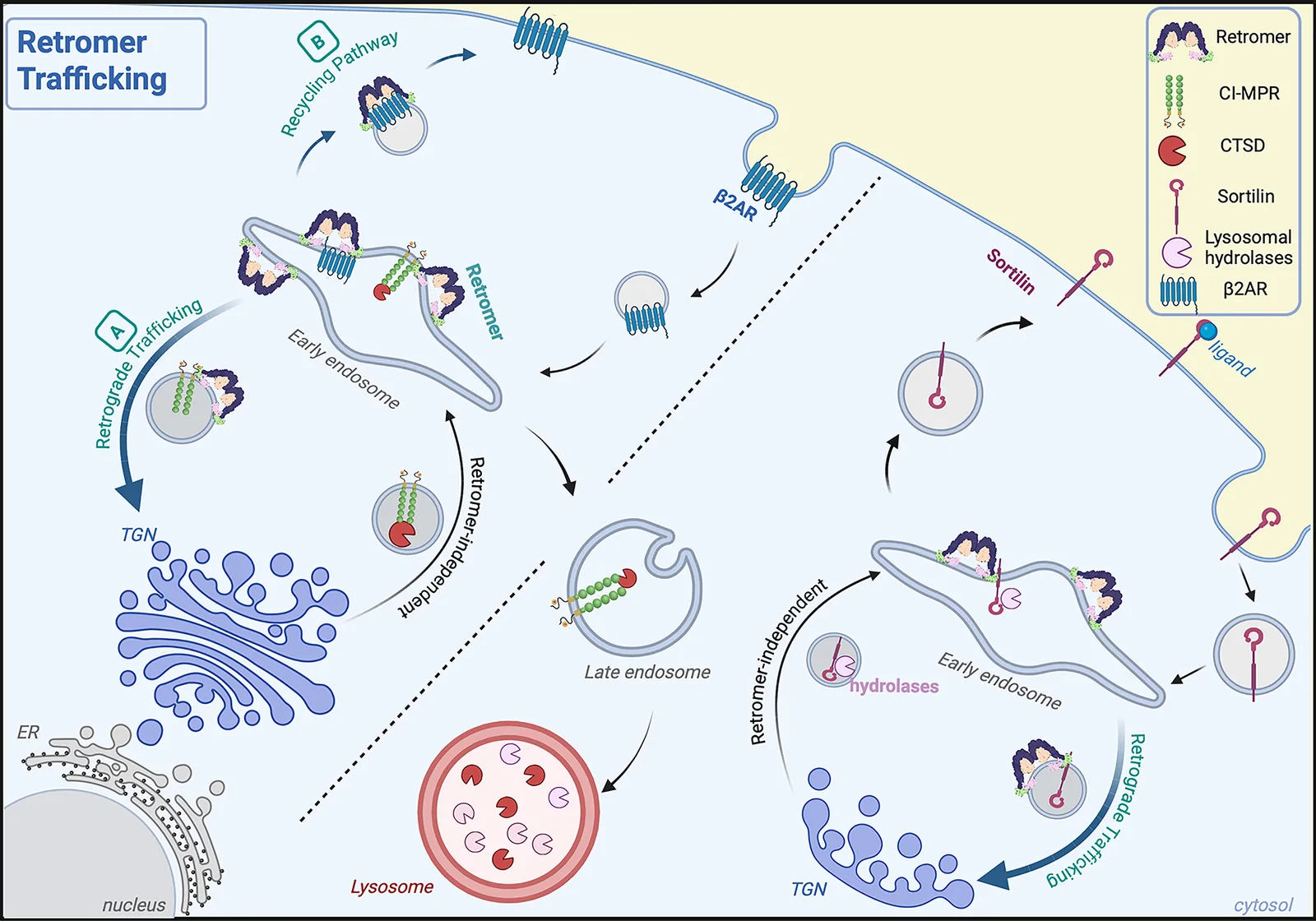
Role of the retromer complex in endosomal protein trafficking and lysosomal homeostasis. Schematic representation of retromer-mediated trafficking pathways within the endolysosomal system. (**A**) Retrograde trafficking: The retromer complex, composed of the core subunits VPS35–VPS26–VPS29, is recruited to early endosomal membranes, where the VPS35 subunit mediates the recognition of selected cargoes, including the cation-independent mannose-6-phosphate receptor (CI-MPR) and Sortilin, that are sorted from early endosomes back to the trans-Golgi network (TGN). This pathway is essential for the proper recycling of CI-MPR and Sortilin, which at the TGN subsequently regulate, through retromer-independent mechanisms, the sorting and trafficking of lysosomal hydrolases, such as cathepsin D (CTSD), through early endosome and late endosomes, ensuring their efficient delivery to lysosomes. (**B**) Endosome-to-plasma membrane recycling pathway: The retromer regulates the retrieval of surface receptors, such as the β2-adrenergic receptor (β2AR), thereby preventing their mis-sorting toward late endosomes and lysosomal degradation. β2AR is recognized by the VPS35 subunit in the early endosomes and sorted to the plasma membrane. β2AR internalization from the plasma membrane to early endosomes is a retromer-independent mechanism. Created in https://BioRender.com

As previously described, the retromer plays a key role in cargo trafficking from the EE to the plasma membrane or the TGN, and from mitochondria to peroxisomes or lysosomes (Fig. 5). The relevance of the retromer in modulating the ELS has been demonstrated in *VPS35* knockout (KO) H4 neuroglioma cells and mouse neurons, which revealed profound changes to the endo-lysosomal network, with hybrid and enlarged endo-lysosomal compartments loaded with undigested intraluminal material, and limited degradative capacity [90]. An integrated multi-omics approach in the same in vitro model revealed significant depletion of lysosomal hydrolases [90]. To follow, a detailed discussion of the ELS mechanisms regulated by the retromer.

*Lysosomal hydrolase sorting: * VPS35 indirectly controls lysosomal activity by transporting lysosomal hydrolase receptors, such as cation-independent 6-mannose phosphate receptor (CI-MPR) and Sortilin (Fig. 5) [104]. These receptors are directly involved in the sorting and subsequent maturation of lysosomal hydrolases. *VPS35* KO reduces lysosomal proteolytic function because of the disrupted processing of lysosomal hydrolases, dependent on the trafficking of the CI-MPR and Sortilin [105–107]. Vps35/Vps26 interacts with Sortilin via the sorting motif YSVL and mutagenesis of this site impairs the binding with Vps35/Vps26 [108]. CI-MPR trafficking can be mediated by the retromer or ESCPE-1 [16, 17]. SNX6, a component of the ESCPE-1 complex, directly interacts with RAB32 through its phox homology (PX) domain and this interaction is linked to the recycling of CI-MPR to the TGN [109]. ESCPE-1 recognizes two antiparallel β-strands, βA and βB, of a β-hairpin-shaped sorting motif in CI-MPR. Through this interaction, ESCPE-1 carries CI-MPR from the endosomes to the TGN [16]. The retromer controls CI-MPR trafficking directly via interaction between the cytosolic domain of CI-MPR and hVPS35 [104], or indirectly via Sortilin-related receptor 1 (SORL1, also known as SORLA), a retromer cargo [110]. SORLA directly binds VPS26 via a phenylalanine residue located in a six-amino acid FANSHY sequence in the SORLA tail [111]. SORL1 levels were found to be decreased in AD brains [112]. In SORL1-deficient human-induced pluripotent stem cell (hiPSC)-derived microglia-like cells, CI-MPR has reduced expression and reduced co-localization with enzymes in lysosomes, highlighting how CI-MPR levels and localization are intertwined with SORL1 [113].

*Retrograde protein trafficking to the TGN: *The retromer-dependent retrograde cargo trafficking requires SNX3 [105]. SNX3 facilitates retromer’s localization to the endosomal membrane [93]. SNX3 predominantly localizes to the early endosomes and is involved in retrograde protein trafficking to the TGN [114]. SNX3 sorting role resides in binding PI3P on early endosomes via the phox-homolog domain [11]. Via this function, SNX3 regulates the transport of different transmembrane proteins such as CI-MPR (Fig. 5), Wnt sorting receptor Wntless, APP, transferrin receptor (TfR) and the iron transporter Fet3/Ftr1 [105, 114–117]. In HEK293T cells, overexpression of SNX3 correlates with decreased APP internalization, higher levels on the plasma membrane and decreased levels of secreted Aβ, which points towards a SNX3-mediated salvage of APP from the intracellular amyloidogenic pathway [115]. The evidence of SNX3 involvement in AD is further strengthened by the identification of SNX3 polymorphisms associated with the risk of AD [93]. Further details of how the SNX3 impacts PD are further discussed in the oxidative stress subparagraph.

*Transmembrane protein sorting to the plasma membrane:* A retromer adaptor relevant in protein sorting to the plasma membrane is SNX27. SNX27 contains an N-terminal domain with PSD95, Dlg1, and zo-1 (PDZ), which binds VPS26A and many synaptic receptors [118, 119]. An exposed β-hairpin in the SNX27 PDZ engages a groove in the arrestin-like structure of VPS26A [118]. Different synaptic receptors interact with SNX27 via the class I PDZ-binding motifs (PDZbms) (S/T-x-Φ) and form a high or low affinity bond [119, 120]. Acidic residues (Asp/Glu) located − 3 and/or − 5 positions upstream of the PDZbms are negatively charged and interact with a positively charged and conserved Arg58 on SNX27, significantly enhancing binding affinity [120]. β_2_ adrenergic receptor (β2AR) and N-methyl-D-aspartate (NMDA) receptors lack these upstream sequences and instead have a conserved phosphorylation site for Ser/Thr residues − 3, −5 or -6 positions upstream of the PDZbms that dramatically enhances interactions with SNX27 [120]. In addition, SNX27 contains the 4.1/ezrin/radixin/moesin (FERM)-like domain, able to bind Ras GTPase and a DLF motif within the N-termini of SNX1/2 [121]. Interestingly, a FERM-like domain is also present on SNX17, a cargo adaptor of the retriever and not of the retromer complex [122]. The FERM domain on SNX17 has been shown to bind the Asn-Pro-Xaa-Tyr (NPxY) sequences on the APP cytoplasmic tail [121]. In contrast, SNX27 does not recycle NPxY-containing cargo, highlighting how the FERM domain on different SNXs mediates distinct endocytic recycling pathways [123].

Through these direct interactions, SNX27 traffics retromer cargoes from the endosome to the plasma membrane in a handover mechanism with ESCPE-1 and indirectly segregates retromer cargo proteins in specific endosomal areas via a FAM21-mediated interaction with the WASH complex [20, 28, 119, 124, 125]. SNX27 is also identified as a regulator of transmembrane protein sorting to the cell membrane via the Golgi-independent unconventional protein secretion under cellular stress [126]. SNX27 modulates unconventional protein secretion along with the VPS26A-containing retromer complex [126]. SNX27 deficiency has been identified in Down syndrome (DS) brains and *Snx27*^*−/−*^ mice presented synaptic and APP processing dysfunction [127, 128]. SNX27’s role in neurodegenerative mechanisms is further explored in the proteostasis and neurotransmission section of this review.

*RAB GTPases regulation:* Endosomal recycling is regulated through an endosomal nanodomain, the retrieval subdomain, physically distinct from the ESCRT-demarcated degradative subdomains [129]. This subdomain integrates cargo recycling with the regulated switching of specific RAB GTPases [129]. The retromer directly associates and recruits the RAB10 regulators DENND4A, DENND4C, TBC1D1 and TBC1D4, the RAB10 effector and RAB35 GTPase-activating protein TBC1D13, and the RAB7 GTPase-activating protein TBC1D5 to regulate the function of the retrieval subdomain [25, 129]. A conserved hydrophobic cavity on VPS29 (defined by Leu152) interacts with Pro-Leu motifs on TBC1D5, DENND4A and DENND4C, whereas VPS35 interacts with an Ile-Pro-Phe motif on TBC1D5 [129]. TBC1D13 envelops VPS29 using an extensive clamp structure composed of two loop sequences and interacts through three notable interfaces [129]. As such, the retromer has been proposed as a mediator for GTPase regulation in the retrieval subdomain [129]. Interestingly, the retrieval subdomain dysfunction is associated with neurodegeneration [74]. Recruitment of RAB7a through the retromer-TBC1D5 complex enables the correct sorting of autophagy-related protein 9a (ATG9a) and autophagosome formation around damaged mitochondria during Parkin-mediated mitophagy [25]. This evidence suggests that the retromer is at the cornerstone between ELS and mitochondrial dynamics. How the retromer-mediated regulation of RAB proteins impacts specific mechanisms of neurodegeneration is discussed in the following paragraphs.

### Retromer-mediated regulation of the endosomal-lysosomal system modulates key neurodegenerative mechanisms

The authors of this review postulate a novel theoretical model of neurodegeneration, in which dysfunctional retromer subunits in AD, PD and ALS are responsible for the impairment of the ELS homeostasis, which in turn, triggers downstream neurodegenerative mechanisms (Fig. 1). As previously stated, in a 2025 large-scale high-throughput proteomic study led by the Global Neurodegeneration Proteomics Consortium, impaired proteostasis, neurotransmission, mitochondrial homeostasis, and neuroinflammation were converging biological pathways across neurodegenerative diseases centered around the ELS (Fig. 1) [87]. To follow is an in-depth discussion of how the retromer affects each one of these mechanisms. In particular, via the regulation of the ELS and endosomal-mediated protein trafficking, the retromer plays a role in (i) proteostasis, protein misfolding and aggregation; (ii) mitochondrial homeostasis via regulation of proteins involved in fusion, fission, mitophagy and mitochondrial-related reactive oxidative species (ROS) production; (iii) synaptic function by controlling synaptic growth, synaptic receptor recycling and neurotransmitter reuptake; (iv) neuroinflammation, by monocyte and microglia regulation (Table 1).

**Table 1:** Molecular mechanisms through which the retromer interacts with different proteins regulating the misfolded protein clearance, mitochondrial biology, neurotransmission, and neuroinflammation in Parkinson’s disease, Alzheimer’s disease, and Amyotrophic Lateral Sclerosis. If a protein is listed next to a specific neurodegenerative disease, it has been studied in pre-clinical models of that specific neurodegenerative condition

Disease	Pathway involved	Molecules involved	Mechanism	References
Alzheimer’s disease	Misfolded protein clearance	APP, tau	VPS35 promotes the clearance of Aβ and tau and reduces APP processing	[89, 90, 96, 138, 147, 148, 154]
	Misfolded protein clearance (ELS)	CI-MPR	VPS35 and VPS26 regulates axonal retrograde transport, impairing lysosomal proteolysis	[153]
	Impaired protein recycling	SORL1 (or SORLA)	VPS26b regulates SORL1-mediated distribution of APP	[94]
	Oxidative stress	Lipid droplets	VPS26 and VPS35-mediated lipid droplets formation, a sequestration site for peroxidized lipids	[198]
	Neurotransmission	GluA1 (or GluR1)	VPS26B more than VPS26a and VPS35 regulates GluA1 trafficking and surface expression, modulating LTP and abundance of dendritic spines	[94, 147, 249, 256]
		NEV	Vps35 modulates APP delivery to synaptic NEVs and NEV trafficking between neurons in *Drosophila*	[241]
	Neuroinflammation	Synaptic proteins	Microglial VPS35 knock-out leads to increased hippocampal synaptic proteins phagocytosis and decreased synaptic density	[298]
		TREM2	VPS35 facilitates TREM2 recycling to the microglia plasma membrane	[282]
		iNOS	VPS35 deficiency leads to LPS-induced iNOS expression and IL-6 production	[282]
		Beclin-1	VPS35 recruitment to phagosomal membrane is regulated by Beclin-1	[281]
		APOE	VPS35 modulates APOE levels	[147]
Parkinson’s Disease	Misfolded protein clearance	αS	Retromer enhancement increases αS clearance	[155, 165, 166]
	Misfolded protein clearance (macroautophagy)	FAM21 (WASH complex subunit)	VPS35 binds FAM21 favoring FAM21 interaction with the WASH complex	[133, 143, 144, 172, 173]
		P62	Retromer enhancement decreases p62 levels	[158]
		LC3-II	Retromer enhancement increases LC3-II levels	[158]
	Misfolded protein clearance (ELS)	LRRK2	Retromer functionally interact with LRRK2, mitigating LRRK2 hyperactivity	[173, 181]
		RAB7a	VPS35 or VPS29 maintains localization of RAB7a and TBC1D5, a RAB7-specific GTPase-activating protein	[25, 129]
		RAB10	VPS35 modulates RAB10 phosphorylation in mice	[99]
		RAB27b	VPS35 modulates RAB27b levels, a regulator of lysosomal exocytosis	[90]
		RAB39b	VPS35 regulates RAB39b levels, which in turn regulates α-Syn accumulation	[90, 184, 185]
		RILPL1	VPS35 regulates recruitment of RILPL1, a RAB effector protein, to the lysosomes	[170]
		Sortilin	Retromer enhancement increases Sortilin levels	[155]
		CI-MPR	Retromer enhancement increases CI-MPR levels	[155]
		CTSD	Retromer enhancement increases the mature form of CTSD	[155]
		CTSL	VPS35^D620N^ is associated with decreased CTSL levels	[169, 170]
	Misfolded protein clearance (CMA)	LAMP2A	VPS35 is required for Endosome-to-Golgi Retrieval of LAMP2A	[134, 155, 158]
		HSC70	VPS35 enhancement is associated with increased HSC70 levels	[155, 158]
	Protein sorting	Parkin	Parkin-mediated polyubiquitination of VPS35 is a regulatory mechanism for retromer-dependent sorting	[216]
	Mitochondrial fusion	MFN2	VPS35 clear MUL1 from the outer mitochondrial membrane, thereby decreasing MUL1-mediated ubiquitination of MFN2	[137]
	Mitochondrial fission	DRP1 (or DLP1)	VPS35 binds Drp-1, assisting in its recycling	[203, 204]
	Micromitophagy	MAPL (or MUL1)	VPS35 and Vps26 regulates trafficking of MDV-related MAPL to peroxisomes	[67, 213, 215]
	Oxidative stress	αS	αS accumulation inhibits SNX3-mediated recycling of iron transporters	[116]
	Mitochondria function (oxidative stress)	PLA2	PLA2 modulates VPS35 and VPS26 function in retrieving ceramides, which interact with complex III of the electron transport chain	[230, 231]
	Neurotransmission	DRD1	VPS35 favors DRD1 localization to cell surface and phosphorylation of the dopamine signaling effectors CREB and ERK	[258]
		DAT	VPS35 binds DAT and interacts with LRRK2 for DAT function modulation and striatal dopaminergic transmission	[261, 262, 299, 300]
		VMAT2	VPS35 directly interacts with VMAT2 and increases VMAT2 levels	[158, 267]
		GluR1 (or GluA1)	VPS35 modulates GluR1 trafficking	[244, 301]
		β2AR	SNX27 controls β2AR downregulation and recycling to the cell surface	[251]
	Neuroinflammation	RAB10	VPS35^D620N^ increased levels of LRRK2-mediated RAB10 phosphorylation in monocytes	[99]
Amyotrophic lateral sclerosis	Misfolded protein clearance (ELS)	SOD1	Retromer enhancement degrade SOD1 by boosting ELS	[103, 292]
		TDP-43 inclusions	Retromer subunits VPS26A, VPS26B, VPS29, VPS35, SNX6, SNX12-mediated ELS sorting regulates TDP-43 inclusion formation and clearance	[101]
		CTSD	Retromer enhancement increases levels of CTSD	[103, 292]
		CI-MPR	Retromer enhancement increases levels of CI-MPR	[103, 292]
		NEK1	NEK1 modulate endosomal trafficking by phosphorylating VPS26B	[302]
	Neurotransmission	GluA1 (or GluR1)	Retromer modulates GluA1 recycling	[102]

*Abbreviations*: αS = α-Synuclein; β2AR = Beta-2 Adrenergic Receptor; Aβ = Amyloid Beta; APOE = Apolipoprotein E; APP = Amyloid Precursor Protein; CI-MPR = Cation-independent Mannose Phosphate Receptor; CTSD = cathepsin D; CTSL = Cathepsin L; DAT = Dopamine Transporter; DRD1 = Dopamine Receptor D1; DLP1 = Dynamin-like protein 1; DRP1 = Dynamin-related Protein 1; FAM21 = Family with sequence similarity 21; GluA1 = Glutamate Receptor Subunit A1; GluR1 = Glutamate Receptor 1; GTPase = Guanosine Triphosphatase; HSC70 = Heat Shock Cognate Protein 70; IL-6 = Interleukin-6; iNOS = inducible Nitric Oxide Synthase; LAMP2A = Lysosomal-Associated Membrane Protein 2 A; LC3-II = Light Chain 3-II; LPS = Lipopolysaccharide; LRRK2 = Leucine-Rich Repeat Kinase 2; LTP = Long-Term Potentiation; MAPL = Mitochondria-Associated Protein Ligase; MFN2 = Mitofusin 2; MUL1 = Mitochondrial E3 Ubiquitin Protein Ligase 1; NEK1 = NIMA-related kinase 1; NEV = Neuronal Extracellular Vesicles; PLA2 = Phospholipase A2; SOD1 = Superoxide Dismutase 1; SORL1 = Sortilin-related receptor 1; TBC1D5 = TBC1 domain family member 5; TDP-43 = Transactivase response DNA-binding protein 43; TREM2 = Triggering Receptor Expressed on Myelod cells; VMAT2 = Vesicular Monoamine Transporter 2; VPS35 = Vacuolar Protein Sorting 35; VPS26a = Vacuolar Protein Sorting 26a; VPS26b = Vacuolar Protein Sorting 26b; WASH = Wiskott-Aldrich Syndrome protein and scar homologue complex

#### Proteostasis, protein misfolding and aggregation

Protein misfolding and aggregation into toxic multimers with loss of the physiological function of the misfolded proteins have been traditional mechanisms proposed for different neurodegenerative diseases, with Aβ and hyperphosphorylated tau for AD, αS for PD, and SOD1 and TDP-43 for ALS [40, 57, 130, 131]. As such, the hypothesis that reducing the amount of these toxic aggregates would be neuroprotective has been the subject of investigations and debate for decades [132].

The retromer has a key role in proteostasis via the endosomal network and the regulation of the trafficking of proteins involved in cellular catabolic pathways, i.e. ELS itself, macroautophagy (MA), chaperone-mediated autophagy (CMA), and the ubiquitin-proteasome system (UPS) [104, 133, 134]. These pathways are intertwined via significant cross-talk, leading to a reciprocal modulation in which the retromer is involved [90, 135]. ELS, MA, CMA, UPS are essential for neuronal proteostasis through the maintenance of a subset of the proteome with a higher risk of misfolding than the general proteome [104, 133, 136–138]. These pathways have been demonstrated to be altered in patient samples or pre-clinical models of ALS, PD and AD, with consequent accumulation of toxic misfolded proteins (Table 1) [139–141].

*Macroautophagy:* VPS35 governs macroautophagy by controlling the trafficking of ATG9A, the only transmembrane protein of growing autophagosomes [142]. The adequate sorting of ATG9A is coordinated by RAB7A, whose functionality is in turn regulated by VPS35 and VPS29 [25]. In addition, VPS35 controls the localization of the WASH complex, a multiprotein structure involved in actin polymerization and consequent endosomal tubulation and scission [143]. The WASH complex is necessary for autophagosome formation [133]. VPS35 governs MA also by controlling the WASH complex’s sorting and recycling via direct interaction with one of its subunits, the protein FAM21 (Fig. 3) [28, 29, 143, 144].

*CMA:* VPS35 directs CMA by sorting lysosome-associated membrane protein 2 A (LAMP2A), a protein receptor located on lysosomes (Fig. 3) [90, 134]. *VPS35* KO cell multi-omics analysis revealed LAMP2 displacement to the cell membrane, which may be secondary to the enhanced lysosomal exocytosis identified in *VPS35* KO cells and highlights the retromer’s role in appropriate LAMP2 positioning on the lysosomal cell membrane [90]. CMA regulates the degradation of cytosolic proteins carrying a CMA recognition motif such as KFERQ. The KFERQ motif is recognized by the chaperone proteins heat shock protein 8/heat shock cognate protein 70 (HSPA8/HSC70), which sorts the substrate to lysosomes (Fig. 3). The substrate binds LAMP2A and the complex is internalized into the lysosomes, where LAMP2A dissociates for recycling, whereas the cytosolic protein will be degraded by lysosomal hydrolases [145].

*UPS:* A significant increase in poly-ubiquitinylated protein levels was identified in *VPS35*-downregulated brain endothelial cells or embryonic neurons, which points towards a regulatory role of the retromer in the UPS system function [138, 146]. A direct or indirect interaction between the retromer and the proteasome has yet to be demonstrated.

*Alzheimer's disease:* There are three mechanisms related to endosomal system dysfunction through which the retromer is involved in amyloid and tau pathology in AD: (i) production of toxic forms of amyloid and tau, (ii) their reduced clearance and (iii) dysfunctional recycling of APP [90, 94, 96, 138, 147, 148].

*Impaired production:* The retromer cargo adaptor SNX27 regulates γ-secretase activity via interaction with the presenilin-1 subunit of this complex in vitro and in vivo [128]. Reduced SNX27 expression leads to markedly increased Aβ generation and reduced APP levels [128]. SNX27 overexpression decreases Aβ generation in the hippocampus of Tg2576 mice harboring the Swedish familial mutation in human *AAP* mice [128]. This evidence highlights SNX27’s role in regulating Aβ generation by modulating γ-secretase activity [128].

*Reduced clearance:* Sortilin and CI-MPR are two retromer cargoes involved in carrying lysosomal hydrolases to the lysosomes (Fig. 5) [149, 150]. CI-MPR is deficient in the AD model of hiPSC-derived microglia-like cells, and decreased expression levels of Sortilin, CI-MPR, cathepsin D (CTSD), VPS35, VPS26, and VPS29 have been detected in the cortex of Tg2576 mice expressing human APP with the Swedish mutation (K670N/M671L) [151, 152]. In AD mouse models, the axonal retrograde transport of VPS35 and VPS26 is impaired, and retromer-mediated CI-MPR retrieval is disrupted, causing protease deficiency in lysosomes and impaired lysosomal proteolysis [153]. Similar findings have yet to be demonstrated for Sortilin in AD preclinical models. The retromer enhancer R55 diminishes Aβ peptide levels, pointing towards a retromer-mediated reduction of APP processing and overall retromer function in limiting the generation of toxic amyloid [154]. The retromer enhancer R33 led to an increase in VPS35, VPS26, and VPS29, CI-MPR, and CTSD levels and to a reduction in beta-amyloid levels, accompanied by improved memory and synaptic integrity [152]. These data suggest the retromer’s role in modulating the clearance of Aβ and tau via the distribution of the lysosomal hydrolases-carrying protein CI-MPR, and lysosomal hydrolases within the ELS system (Fig. 2). In vivo and in vitro ALS and PD models manipulated with retromer enhancers registered an increase in levels of the mature form of CTSD [103, 155]. Further studies are needed to investigate the relationship between the retromer and mature forms of cathepsins in AD.

In AD mouse models, CMA plays a key role in maintaining neuronal proteostasis of the proteome subset with a higher risk of misfolding [136]. Tau and APP are potential targets for CMA-mediated clearance [156, 157]. At their C-terminus, tau has multiple KFERQ-like motifs, and APP contains one KFERQ motif [156, 157]. HSC70, a key mediator of CMA, binds the KFERQ recognition motif [156]. Early evidence in PD revealed that VPS35 enhancement is associated with increased HSC70 levels [155, 158], though this has yet to be demonstrated in AD models.

*Dysfunctional recycling:* SORL1 is involved in the sorting of APP from the endosomes to the TGN and plasma membrane, preventing amyloidogenic processing (Fig. 2) [159, 160]. In SORL1-deficient hiPSC-derived microglia-like cells, amyloid beta 1–42 accumulates [113]. SORL1 exerts its sorting effect via interaction with Vps26b [159]. Silencing *Vps26b* in mice significantly decreases SORL1 levels in the plasma membrane and increases Aβ and tau accumulation in brain tissue and CSF [94]. These data suggest how impaired APP recycling secondary to the ELS dysfunction can be a pathogenetic mechanism of AD.

*Parkinson’s disease:* Emerging data have highlighted the MA, ELS, and CMA contributions to αS degradation (Fig. 3) [161–164]. In transgenic mice overexpressing human αS, the lentiviral-delivered expression of human VPS35 rescues αS-induced neurodegeneration [107]. Similarly, retromer enhancement with the molecular chaperone 1,3 bis-phenyl guanylhydrazone (or 2a) protects against neurodegeneration and favors αS clearance in a PD mouse model, potentially due to the upregulation of the αS degradation pathways, CMA, ELS, and MA [155, 165, 166]. In in vivo and in vitro models of PD, 2a increases the levels of LAMP2A, HSC70, markers of MA, and favors the endosome-to-Golgi retrieval of LAMP2A, increases light chain 3-II (LC3-II) and decreases p62 (an αS receptor), indicating enhanced MA flux [167, 168], increases levels of the mature forms of CTSD, modulates cathepsin L (CTSL) levels and lysosomal hydrolases carriers Sortilin and CI-MPR, indicating an enhanced lysosomal activity, with subsequent reduction in pathological αS levels [134, 155, 158, 166, 169, 170]. Pharmacological inhibition of LRRK2 in *VPS35*^*D620N*^ cells did not affect CTSL levels [170], highlighting the retromer-dependent modulation of CTSL levels. Of note, αS carries the KFERQ recognition motif recognized by the chaperone proteins HSPA8/HSC70, which sorts the substrate to LAMP2A, favoring αS degradation via CMA (Fig. 3) [171]. Retromer binding to FAM21 and the WASH complex is perturbed by the PD-linked *VPS35*^D620N^ mutation, with VPS3*5*^D620N^ forming clusters with FAM21 in EEs and disrupting MA (Fig. 3) [133, 172, 173]. The retromer governs ELS also by its interaction with RAB proteins and LRRK2, whose mutations have been associated with familial PD [174–176]. LRRK2 inhibits the lysosomal degradative activity, as well as regulates proper vesicular transport and autophagy via interaction with RAB GTPases, among which RAB10 [177–179]. VPS35^D620N^ recruits and activates LRRK2 on the lysosomal surface, driving assembly of the RILPL1-TMEM55B complex, which coordinates autophagosome formation and lysosomal repair [170, 180]. VPS35 can protect against neuronal death caused by LRRK2 mutations, suggesting that VPS35 overexpression may mitigate some of the toxic effects associated with LRRK2 hyperactivity via a functional interaction [181] and rescue the neurodegenerative phenotype induced by LRRK2 [177]. VPS35 modulates the levels of RAB27b [90], a modulator of lysosomal exocytosis and cell-to-cell transmission of pathogenic αS aggregates [182, 183] and of RAB39b, a regulator of αS accumulation [90, 184, 185].

However, Chen et al. found that VPS35 depletion fails to increase levels of endogenous αS protein in SH-SY5Y cells and that genetic overexpression of wild-type VPS35 in Sprague-Dawley rats does not prevent αS accumulation, nor protect against αS-induced nigral dopaminergic neurodegeneration [186]. This evidence contrasts with the prior body of evidence highlighting that *VPS35* loss of function enhances αS accumulation within the lysosomes, and αS toxicity in yeast, worms, and mouse and emerging evidence highlighting 2a-mediated enhancement of VPS35 function protects against neurodegeneration and favors αS clearance [107, 187]. Differences in these results may reflect different retromer manipulation methods, i.e. genetic overexpression versus pharmacological modulation. While genetic approaches may induce sustained and potentially supraphysiological increases in protein expression, pharmacological modulation may achieve a more controlled enhancement of retromer activity, potentially leading to different retromer functional outcomes and cellular responses. Second, retromer structure depends on the coordinated assembly of multiple subunits [188]. Disproportionate changes in concentration of individual subunits could disrupt complex stability, leading to aberrant protein interactions and impaired retromer functionality [104]. In this context, it is relevant that 2a increases the levels of all the subunits of the retromer, VPS35, VPS26b and VPS29 subunits, whereas in Chen et al. investigation, selective genetic overexpression of VPS35 was implemented [103, 186]. Thus, while moderate and balanced enhancement of retromer component expression may promote retromer activity, excessive and/or unbalanced overexpression may paradoxically compromise retromer function. To address these questions, it will be essential to evaluate whether genetic overexpression effectively enhances retromer function by assessing the trafficking, localization, and protein levels of established retromer cargoes and lysosomal hydrolases, including CI-MPR, LAMP2A, and CTSD. These analyses will help determine whether the intervention improves endosomal–lysosomal trafficking and cargo recycling in a functionally meaningful manner.

*Amyotrophic Lateral Sclerosis:* Similarly, in ALS mouse models, the retromer enhancer 2a increases the levels of mature CTSD and CI-MPR, reduces the levels of misfolded SOD1, and rescues motor neurons in a SOD1 mouse model, highlighting that the retromer protects against neurodegeneration by boosting SOD degradation via the ELS [103]. In ALS MNs, declining expression of retromer components (VPS26A, VPS26B, VPS29, VPS35), SNX6 and SNX12 accompanies increasing presence of TDP-43 + cytoplasmic inclusions, which supports the hypothesis that retromer complex-mediated endolysosomal sorting is involved in the formation or clearance of TDP-43 inclusions (Fig. 4) [101]. Impaired TDP-43 clearance leads to its mislocalization within neurons and subsequent alternative polyadenylation of the 3’ untranslated region of the *VPS35* transcript [189]. This is associated with reduced VPS35 and VPS29 protein expression, which in turn leads to ELS dysfunction, TDP-43 accumulation and hyperphosphorylation [189]. This mechanism has been demonstrated in patients with frontotemporal dementia [189], a neurodegenerative disorder with significant genetic and clinical overlap with ALS [190]. The impairment of an upstream retromer and endosomal trafficking regulator, NIMA-related kinase 1 (NEK1), whose mutation is associated with ~ 3% of ALS cases, reiterates the key role the retromer plays in modulating the ELS in the context of ALS pathophysiology (Fig. 4) [191].

Overall, this retromer function is of potential relevance to the treatment of neurodegenerative diseases, characterized by the accumulation of misfolded, toxic proteins. Pharmacological enhancement or genetic overexpression of the retromer cargo-binding subunit VPS35 has been shown to boost these misfolded protein degradation pathways, decreasing the levels of misfolded proteins and favoring the rescue of the neurodegenerative processes [103, 152, 155, 158]. As such, retromer enhancement may represent a novel therapeutic strategy for the clearance of toxic aggregates typical of these neurodegenerative diseases and a common approach based on overlapping pathogenetic mechanisms. The effect of retromer enhancement to increase the levels of cellular chaperones such as HSC70, which also have a role in reverting the misfolded state [192], may represent an additional strategy through which retromer enhancement can impact neurodegeneration, though this still needs to be investigated.

#### Mitochondrial homeostasis, biogenesis, and oxidative stress

**Fig. 6:**
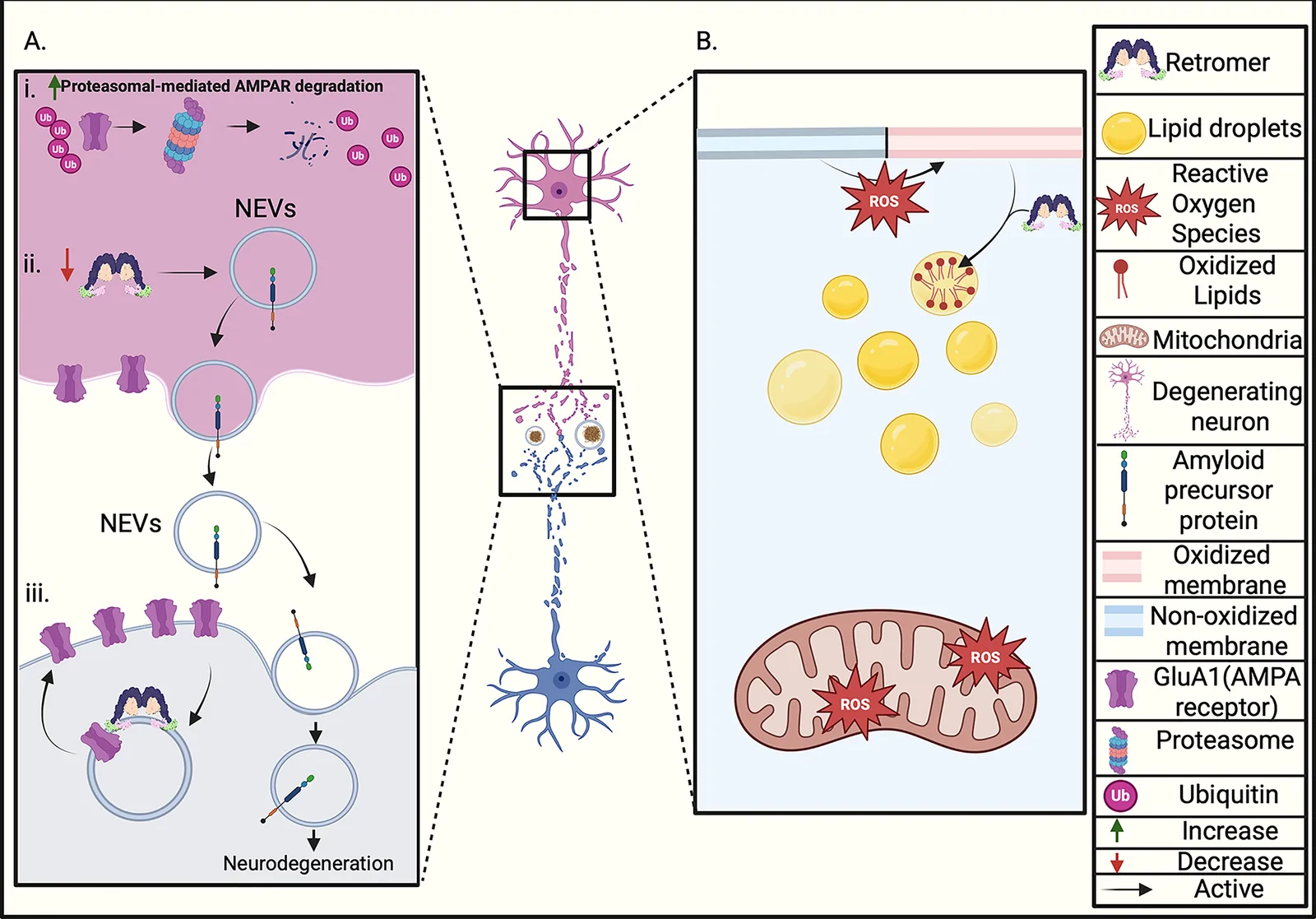
Retromer role in neurotransmission and oxidative stress in Alzheimer’s disease (AD). (**A**) Neurotransmission: (i) Decrease in AMPA receptor (AMPAR) levels and increased AMPA ubiquitination, with consequent neuronal internalization and proteasomal degradation; (ii) Retromer loss leads to increased release of cargoes, such as Amyloid Precursor Proteins (APP), in Neuronal Extracellular Vesicles (NEVs) due to cargo accumulation at the motor neuron presynaptic terminals. NEVs are released by neurons; (iii) The retromer is responsible for the trafficking and surface recycling of Glutamate Receptor Subunit A1 (GluA1). (**B**) Oxidative stress: retromer favors glial lipid droplets formation, a sequestration site for peroxidized lipids, which are a typical mediator of ROS-induced cell injury. Created in https://BioRender.com

Mitochondrial homeostasis, the equilibrium between mitochondrial fusion and fission, and the oxidative reaction chain can be disrupted in PD, AD, and ALS [193]. The retromer modulates mitochondrial fusion, fission, mitophagy, mitochondrial-related ROS production, dampens ROS-mediated damage and prevents apoptosis (Table 1) [194–197]. Overall, the preclinical data on the retromer’s involvement in mitochondrial functionality in AD mainly relate to its role in modulating oxidative stress and oxidative stress-related damage by favoring glial lipid droplets (LD) formation, a sequestration site for peroxidized lipids (Fig. 6) [198, 199]. Despite evidence of altered mitochondrial activity in ALS, further studies are necessary to investigate whether retromer-mediated modulation plays a role in this feature.

*Parkinson’s disease:* Dopaminergic neurons of idiopathic PD patients demonstrate an impaired ability to turn over mitochondria and maintain mitochondrial proteostasis due to alterations in mitochondrial quality control proteins, such as parkin, PTEN-induced putative kinase 1 (PINK1), dynamin-related protein 1 (DRP1), and mitofusin 2 (MNF2) [200]. This may exacerbate the impact of oxidative phosphorylation defects and oxidative stress, contributing to neuronal degeneration [200]. The retromer is a critical player in mitochondrial quality control protein pathways.

*Fusion and fission:* VPS35 gene deletion in dopamine (DA) neurons results in mitochondrial fragmentation, highlighting altered processes of fusion and fission [137]. MFN2 and DRP1 are involved in fusion and fission, respectively.

**Fig. 7:**
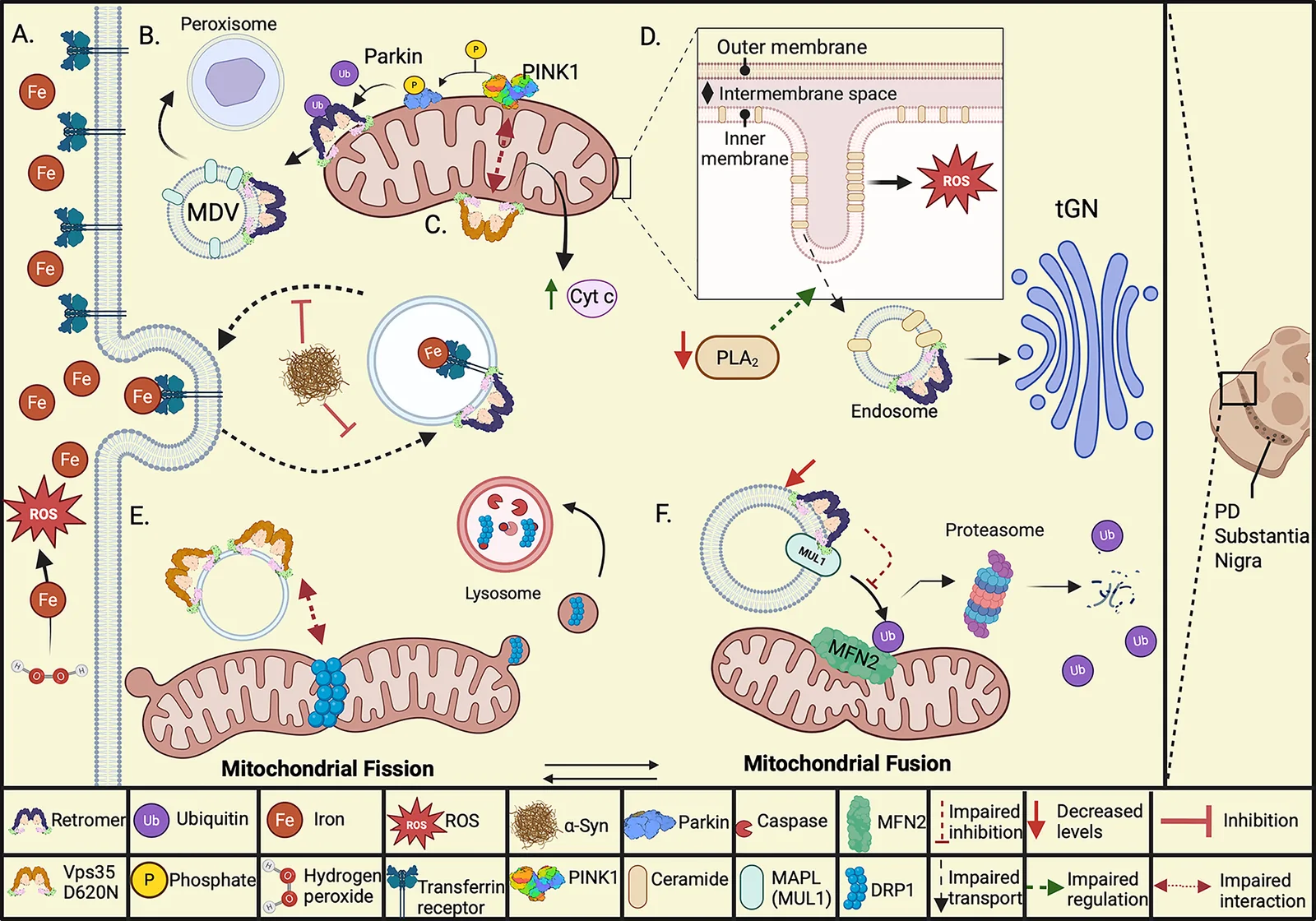
Retromer role in mitochondrial homeostasis and function in Parkinson’s disease substantia nigra. (**A**) Oxidative stress: Ferrous (Fe^2+^) iron attracts one electron from hydrogen peroxide (H_2_O_2_), with consequent H_2_O_2_ monovalent reduction and the production of reactive oxygen species (ROS). Fe^2+^ can be internalized by transferrin receptors, limiting its conversion into ROS. α-Synuclein inhibits transferrin receptor recycling to and from the plasma membrane, whereas the retromer facilitates this recycling. (**B**) Micromitophagy: Parkin phosphorylation by PINK1 increases parkin E3 ligase activity, leading to ubiquitination by parkin of Vps35 and formation of MDVs, which carry damaged and oxidized cargo peroxysomes. VPS35 is recruited to budding MDVs, which are rich in MAPL (also known as MUL1) and modulates MAPL trafficking to peroxisomes. (**C**) VPS35^D620N^ leads to increased release of cytosolic cytochrome c and decreased interaction with PINK1. (**D**) Oxidative stress: Loss of PLA2 stabilizes the retromer and impairs the retrieval of ceramide phosphoethanolamine and sphingomyelin from the mitochondrial membrane to the trans-Golgi network. This leads to an increase in inner mitochondrial membrane ceramides. Ceramides interact with complex III of the electron transport chain, leading to the generation of ROS. (**E**) Mitochondrial fission: Dynamin-related protein 1 (DRP1) assembles into a ring structure on the mitochondrial outer membrane, inducing mitochondrial fission. VPS35 binds DRP1 and assists in DRP1 recycling. The D620N *VPS35* mutation reduced DRP1-VPS35 interaction. (**F**) Mitochondrial fusion: Mitofusin 2 (MFN2) is ubiquitinated for proteasomal degradation by MUL1, promoting mitochondrial fusion. VPS35 deficiency increased Mitochondrial E3 Ubiquitin Protein Ligase (MUL1), leading to MFN2 degradation. Created in https://BioRender.com

Mitochondrial fusion involves the activity of two main proteins, MFN1 and MFN2 and optic atrophy 1 (OPA1) proteins [201]. MFN2 is ubiquitinated for proteasomal degradation by the mitochondrial E3-ubiquitin ligase-1 (MUL1). By promoting the degradation of MUL1, the VPS35 promotes mitochondrial fusion [137] (Fig. 7). VPS35 deficiency increased MUL1, leading to MFN2 degradation in DA neurons (Fig. 7) [137].

Mitochondrial fission requires DRP1, also known as dynamin-like protein 1 (DLP1). DRP1 recruited to the mitochondrial outer membrane (MOM) assembles into a ring structure, which induces fission of mitochondria (Fig. 7) [202]. Interestingly, the carboxyl region of DRP1 contains a short phenylalanine-leucine-valine (FLV) amino acid sequence required for VPS35 binding, which has similarities to known retromer sorting motifs [203]. The VPS35 regulates mitochondrial fission by carrying DRP1 to the lysosome for degradation via an MDV-dependent route (Fig. 7) [203, 204]. The D620N VPS35 mutation reduced DLP1-VPS35 interaction and increased mitochondrial fragmentation (Fig. 7) [203, 204].

*Mitophagy:* Mitophagy is a specialized form of autophagy that regulates the turnover of damaged and dysfunctional mitochondria. Mitophagy uses ubiquitin-dependent (e.g., the PINK1-PRKN pathway) and -independent (e.g., BNIP3L/NIX, FUNDC1, and BNIP3) pathways that converge on the final common autophagic pathway. The PARK2 and PINK1 genes, whose mutations are associated with autosomal recessive juvenile PD [205–207], encode parkin, an E3-ubiquitin ligase [208, 209], and the kinase PINK1. Parkin and PINK1 are involved in a ubiquitin-dependent mitophagy pathway. Upon mitochondrial damage, PINK1 accumulates on the MOM, phosphorylates adjacent ubiquitin on MOM proteins, acting as a receptor for parkin [210]. Once recruited, parkin phosphorylation by PINK1 increases parkin E3 ligase activity, leading to ubiquitination by parkin of proteins on the MOM and initiation of mitophagy through engulfment in isolation membranes that then fuse with lysosomes [211].

Micromitophagy is a selective form of mitophagy of just a mitochondrial fragment, is activated by the PINK1-PRKN pathway and involves MDVs, which carry damaged and oxidized mitochondrial cargo to lysosomes and peroxysomes (Fig. 7) [67, 210, 212, 213]. MDVs formation is independent of the classic protein involved in mitochondrial fission and mitophagy, DRP1 [214]. Budding MDVs are rich in mitochondria-associated protein ligase (MAPL, also known as MUL1), a SUMO E3-ligase whose RING finger regulates mitochondrial morphology by inducing mitochondrial fragmentation if MAPL is overexpressed [215]. Live-cell microscopic analysis revealed that VPS35 is recruited to MDVs and VPS26A and VPS35 modulate MAPL trafficking to peroxisomes (Fig. 7) [67, 215]. The mechanisms by which the retromer is recruited to the MDVs and how its function is modulated to allow a directional transfer of MAPL are unclear. VPS35 works downstream of parkin, and parkin mediates the polyubiquitination of VPS35, which is suspected to serve as a regulatory mechanism for retromer-dependent sorting (Fig. 7) [216]. This evidence suggests that parkin could regulate retromer-mediated MDVs trafficking in mitophagy. On the other hand, in *Drosophila* models, *vps35* interacts genetically with *parkin*, but not *pink1*, and *vps35* overexpression rescues parkin-mutant phenotypes [217]. A loss-of-function mechanism involving parkin and mitochondrial dysfunction may contribute to how the *vps35*^*D620N*^ mutation leads to a PD phenotype [217].

A novel function for MAPL in peroxisomes is the regulation of peroxisome elongation and morphology [215]. However, further studies are required to unravel if the crosstalk between the retromer and mitochondria via MAPL could signify an indirect effect of the retromer on peroxisome function.

*Oxidative stress:* Mitochondria-mediated ROS production is related to electron transport chain activity during oxidative phosphorylation [218]. A leading model describes excessive ROS as the unfortunate byproduct of dysfunctional mitochondria in neurodegenerative diseases [218, 219]. ROS induce cellular damage by modifying the structure of proteins, lipids, carbohydrates and nucleic acid, including mitochondrial DNA (mtDNA) [197, 220]. *Vps35* expression restores mtDNA homoplasmy and alleviates associated defects in a *Drosophila* model carrying a long deletion of mtDNA [197]. Other than mitigating ROS-mediated cellular damage, the retromer also modulates mitochondrial-mediated ROS production [196]. VPS35 underlies a ROS-sensing pathway that lowers mitochondrial translation to decrease ROS levels [196]. One of these pathways includes the transporter solute carrier family 7 member 1 (SLC7A1) [196]. SLC7A1 is an Arg/Lys amino acid transporter localized on the plasma membrane and responsible for the availability of the amino acid arginine, which influences mitochondrial-tRNA (mt-tRNA) charging and consequently, mitochondrial translation [196]. Twenty-two species of mt-tRNAs encoded in mtDNA translate essential subunits of the respiratory chain complexes, which in turn are responsible for ROS production [218, 221]. SLC7A1 localization and function are regulated by VPS35 [196]. Intracellular H2O2 oxidizes cysteine residues in VPS35, blocking plasma membrane localization of SLC7A1, decreasing mitochondrial translation and cellular ROS levels [196].

Stem cell-derived neurons from fibroblasts of an affected p.D620N carrier present decreased mitochondrial membrane potential and respiration, and increased production of ROS and cytosolic cytochrome c (Fig. 7) [222]. Similar evidence has been identified in mouse models, with increased levels of mitochondrial ROS, oxidative stress, and apoptotic markers found in the SN of 16-month-old Vps35^D620^ KI mice [223]. MPTP (1-methyl-4-phenyl-1,2,3,6-tetrahydropyridine), a prodrug of the neurotoxin MPP^+,^ which induces PD-like pathology by causing cell death in the SN dopaminergic neurons, leads to cell injury via mitochondrial complex I inhibition and subsequent oxidative stress [224]. Increased VPS35 expression protects dopaminergic neurons against MPP+ cytotoxicity. This neuroprotection function is partially inhibited by the presence of the PD-associated *Vps35*^*D620N*^ mutation [225]. Together, this evidence suggests a role for the non-mutated retromer in protecting against ROS and oxidative stress.

Iron (Fe) overload can lead to oxidative stress (Fig. 7) [226]. Iron levels are increased in the SN of postmortem PD brain [227]. A decrease in iron uptake due to dysfunctional transferrin receptor (TfR) could explain its accumulation in the SN of PD patients and mice [228] (Fig. 7). SNX3 and VPS35 interact with the TfR to sort it to recycling endosomes (Fig. 7) [117]. αS accumulation inhibits SNX3-mediated recycling of iron transporters in *Saccharomyces cerevisiae* and *Caenorhabditis elegans* (Fig. 7) [116]. This evidence suggests that αS plays a role as a trigger for altered retromer function related to iron metabolism in PD patients, leading to consequent oxidative stress, neuronal injury, and neuronal death.

Under physiological conditions, mitochondria can repair ROS damage to mitochondrial membrane lipids in part via a deacylation-acylation cycle mediated by phospholipase A2 (PLA2) [229]. Loss of PLA2 disrupts retromer function, destabilizing VPS35 and VPS26 and impairing retrieval of ceramide phosphoethanolamine and sphingomyelin (Fig. 7) [230]. This leads to an increase in membrane ceramides (Fig. 7) [230]. Ceramides interact with complex III of the electron transport chain, leading to the generation of ROS, and inducing cellular oxidative stress that causes slow, progressive neurodegeneration (Fig. 7) [230, 231].

*Apoptosis:* The retromer enables cell survival by preventing apoptosis. Apoptosis can be triggered by two main pathways, the intrinsic and the extrinsic pathways, and mitochondria are the main actors of the intrinsic pathway [232]. Following MOM permeabilization, cytochrome *c* is released into the cytosol, where it engages the formation of the apoptosome that leads to activation of the caspase cascade [233]. By facilitating the transport of BCL-XL, an important antiapoptotic protein, to the MOM, the VPS35 avoids MOM permeabilization and potentially avoids the release of cytochrome *c* within the cytosol [234]. This may account for why cells with VPS35 knockdown are more prone to apoptosis [234].

#### Neurotransmission

Neurotransmission impairment may play an important role in the pathogenesis of ALS, PD, and AD. Dopaminergic transmission is affected in PD [40]. Glutamatergic excitotoxicity and degeneration of cholinergic neurons are pathological processes of both AD and ALS [235–237]. The retromer is present at both the neuromuscular junction pre- and post-synapse and regulates: (i) synaptic terminal growth, (ii) neurotransmitter receptor recycling, (iii) neurotransmitter reuptake in the synaptic cleft (Table 1) [146, 217, 238–241].

VPS35 loss causes synaptic terminal overgrowth, increased satellite bouton number and branching formation [217, 239–242]. The VPS35 limits synaptic growth by coordinating with TBC1D5 to regulate RAB7 activity [239]. TBC1D5 is recruited to the retromer through binding to VPS29 [243], a process that is impaired in the VPS29 L152E mutant [25]. RAB7 is dysregulated in VPS35 KO cell lines and accumulates on endo-lysosomal membranes [90]. Disruption of TBC1D5 interactions with RAB7 and the VPS35 leads to presynaptic excessive satellite boutons and branch formation, which phenocopies the loss of TBC1D5 [239]. Additionally, in cortical neurons expressing *VPS35 p.D620N*, p.D620N mutant VPS35 was found in spines ∼30% less frequently compared to wild-type VPS35 in VPS35 wild-type cortical neurons, and a reduced number of dendritic spines was detected [244].

The retromer influences neuroexcitatory chemical synapses through synaptic receptor recycling. In *Drosophila* models underexpressing *vps29*, the ELS was dysfunctional with impairment of synaptic receptor recycling and neurotransmission [245]. The retromer cargo adaptor SNX27 binds PDZ sequences present on many types of receptors, including NMDA receptors (NMDARs), β2AR, and 5-hydroxytryptamine 4a receptors (5-HT_4(a)_Rs) [119, 120, 127, 246–249]. In contrast to prior evidence, Clairfeuille et al. demonstrated that SNX27 does not directly bind the α-amino-3-hydroxy-5-methyl-4-isoxazolepropionic acid receptor **(**AMPAR) [120]. Instead, SNX27 associates with the carboxy-terminal PDZ binding motif of the leucine-rich repeat and fibronectin type-III domain containing protein 2 (LRF2), whose N-terminal region binds AMPAR and regulates AMPAR sorting to the plasma membrane [250]. SNX27 also mediates endosome-to-plasma membrane trafficking of β2AR (Fig. 5) [251]. Considering the role of SNX27 in the trafficking of retromer cargoes from the endosome to the plasma membrane, it can be postulated that it is through SNX27 that the retromer plays a part in regulating synaptic receptor recycling. In DS, chromosome 21 trisomy drives overexpression of miR-155, which leads to decreased *SNX27* expression. *SNX27* loss, in turn, leads to NMDA and AMPA receptor dysfunction associated with DS. Importantly, mouse models suggest that the synaptic and cognitive phenotypes associated with DS can be rescued through *SNX27* overexpression [127].

*Alzheimer’s disease:* In AD, overactivation of NMDAR by glutamate is responsible for calcium-induced neuronal excitotoxicity and consequent loss of cholinergic neurons of the Nucleus basalis of Meynert, with consequent lower cerebral levels of Ach [252, 253]. NMDARs activity is modulated by AMPAR, which can increase or decrease NMDAR activity through sodium cascades or a calcium-calmodulin-dependent inactivation process [254]. In AD patients, there is a decrease in AMPA receptor levels and increased AMPA ubiquitination, with consequent neuronal internalization and proteasomal degradation [255]. The VPS26b and VPS35 modulate the activity and normal neuronal distribution and trafficking of AMPA receptors, controlling long-term potentiation (LTP), the neurobiological process of memory and learning (Fig. 6) [94, 249]. VPS26b and VPS35 deficiency lead to decreased dendritic spines and reduced GluA1 levels in hippocampal neurons and LTP dysfunction in mice [94, 147, 256]. In the 5xFAD mice model of AD, transcranial injection of the retromer enhancer R55 ameliorates synaptic and LTP dysfunction observed in 5xFAD mice [257].

**Fig. 8:**
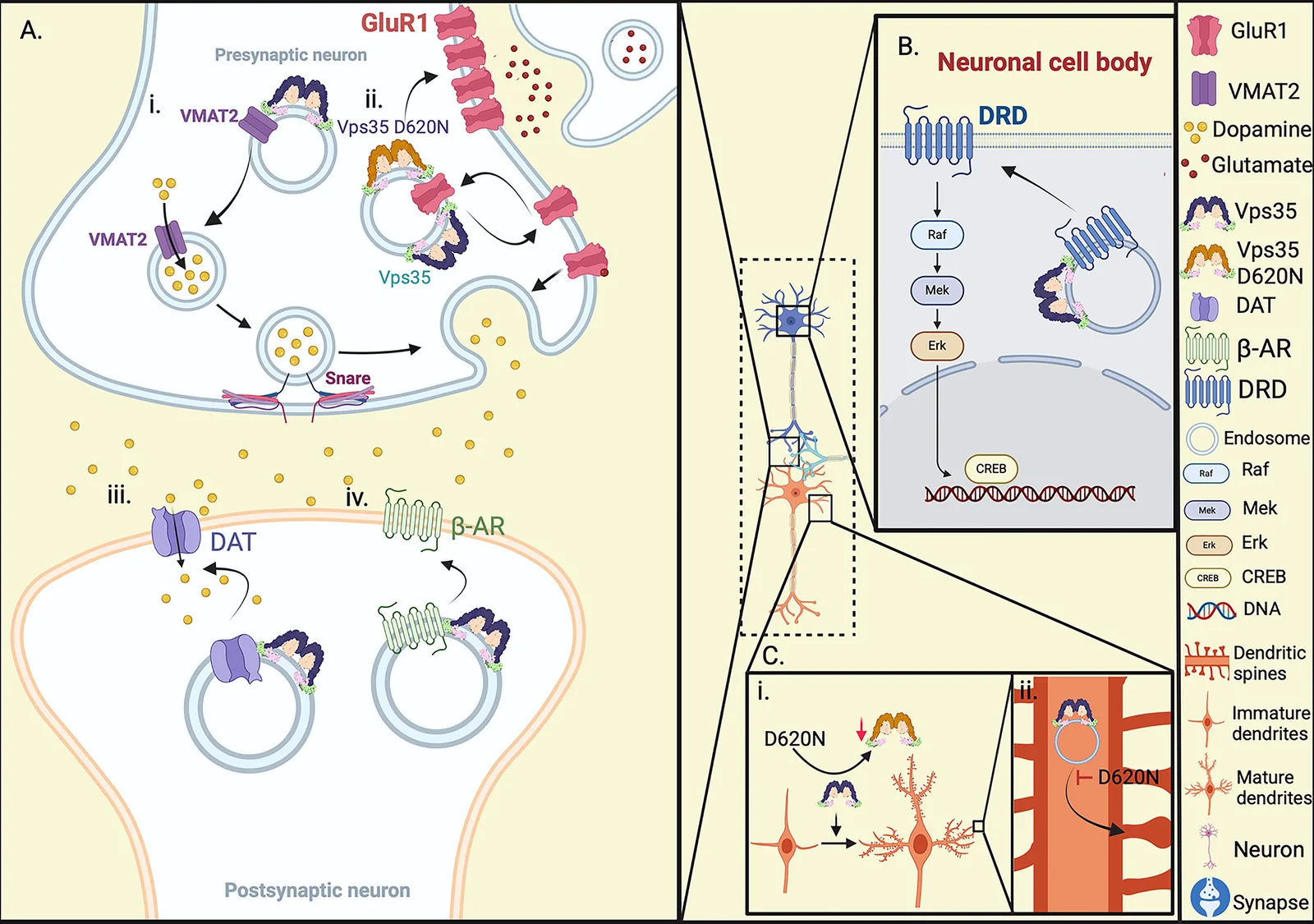
Retromer role in neurotransmission in Parkinson’s disease. (**A**) Retromer’s role at the synaptic junction: (i) Vesicular Monoamine Transporter 2 (VMAT2) is a retromer cargo to the pre-synaptic endosomes; (ii) VPS35^D620N^ neurons reveal re-distribution of Glutamate Receptor Subunit A1 (GluA1, or GluR1), with significantly increased GluR1 cluster intensity, indicating a role for the retromer in GluR1 trafficking between pre-synaptic endosomes and plasma membrane. Glutamatergic synapses on dopaminergic neurons regulate dopamine release via GluR1; (iii) retromer controls Dopamine Transporter (DAT) and (iv) Beta-2 Adrenergic Receptor (β2AR) recycling to the post-synaptic plasma membrane. (**B**) At the neuronal cell body, the retromer regulates surface levels of Dopamine Receptor D1 (DRD1) and phosphorylation of the dopamine signaling effectors CREB and ERK. (C) The D620N mutated form of VPS35 is associated with (i) decreased VPS35 protein levels and impaired dendritic spine maturation with a reduced number of dendritic spines, (ii) localization in dendritic spines ∼30% less frequently compared to its wild-type form. Created in https://BioRender.com

*Parkinson’s disease:* In PD, dopamine receptor D1 (DRD1) and dopamine transporter (DAT) levels are decreased on the synaptic membrane, their trafficking is dysregulated and less DA reuptake results in increased DA metabolite production, such as DOPAC, with consequent oxidative stress and neurodegeneration [137, 258–260]. VPS35 favors DRD1 and DAT recycling to the cell surface, and activation of DRD downstream pathways, as evidenced by phosphorylation of the DA signaling effectors ERK and CREB (Fig. 8) [258, 261].

VPS35 regulates DAT function and striatal DA transmission by functional interaction with LRRK2 [262]. VPS35 could directly interact with LRRK2, as demonstrated by one study revealing LRRK2 and VPS35 co-immunoprecipitation [99], though this was not observed in other studies [242, 263, 264]. Overall, the mechanisms through which VPS35 modulates LRRK2 remain elusive. Vesicular monoamine transporter 2 (VMAT2) is responsible for transporting neurotransmitters from the cytosol into synaptic vesicles. VMAT2 levels are decreased in PD and are associated with nigrostriatal dopamine dysfunction, resulting in neurodegeneration [265, 266]. VMAT is a retromer cargo protein via VPS35 interaction (Fig. 8) [267]. Retromer enhancement increases VMAT2 levels in pre-clinical models of PD [158]. As such, this therapeutic approach could play a role in regularizing dopaminergic neurotransmission in PD via restoring VMAT2 reserves.

Additionally, in PD, GluR1 (also known as GluA1) trafficking is altered, and VPS35 favors appropriate GluR1 re-distribution (Fig. 8) [244]. Given that glutamatergic signaling stimulates DA release from SN, VPS35 indirectly favors appropriate dopaminergic neurotransmission in PD via GluR1 modulation [244].

Recent evidence highlights the role of adrenergic receptor blockers as among the strongest independent factors for an older age of onset of PD [268]. However, other data suggest that beta blockers may be associated with an increased risk of developing PD [269, 270], although these data are controversial [271, 272]. SNX27 controls the downregulation and recycling to the cell surface of β2AR (Figs. 5 and 8) [251]. These early findings highlight a potential new avenue of PD research, in which the retromer-mediated impact on βAR recycling and noradrenergic transmission may play a role in PD age of onset and progression.

#### Amyotrophic Lateral Sclerosis:

In ALS, reduced levels of AMPA subunit GluA1 have been detected, and retromer involvement in AMPAR recycling in motor neurons is supported by early evidence, though yet to be demonstrated in detail (Fig. 4) [102].

In addition to its role in regulating neurotransmitter receptor recycling, the retromer is also involved in the biology of neuronal extracellular vesicles (NEVs). NEVs are released by neurons and glial cells and play a role in intercellular communication in the CNS (Fig. 6) [273]. NEVs also carry biomolecules for degradation, including misfolded forms of proteins involved in neurodegenerative diseases [274]. In AD, increased APP is released extra-cellularly by neurons and spread through neuronal extracellular vesicles (NEVs) [241, 273–275]. Early evidence reveals a role for Vps35 as an upstream regulator of NEV trafficking between neurons and APP delivery to synaptic NEVs in *Drosophila* models [241]. APP is required in both pre and post-synaptic terminals to regulate synaptic function and structure, highlighting how an appropriate APP localization and trafficking are relevant for synaptic physiology [276]. As such, Vps35 may regulate synaptic function via APP trafficking through NEVs, though this has yet to be demonstrated.

Lysosomal exocytosis represents an additional mechanism for extracellular release of pathogenic proteins, including αS, potentially contributing to disease propagation [277]. This has been noted in the context of dysfunctional lysosomes in Tg-αS^A53T^/CSPα^−/−^ mice [277]. However, the role of the retromer complex in this mechanism remains to be established.

#### Neuroinflammation

Neuroinflammation via monocyte and microglia regulation is an additional pathogenetic process in neurodegenerative diseases controlled by the retromer (Table 1).

**Fig. 9:**
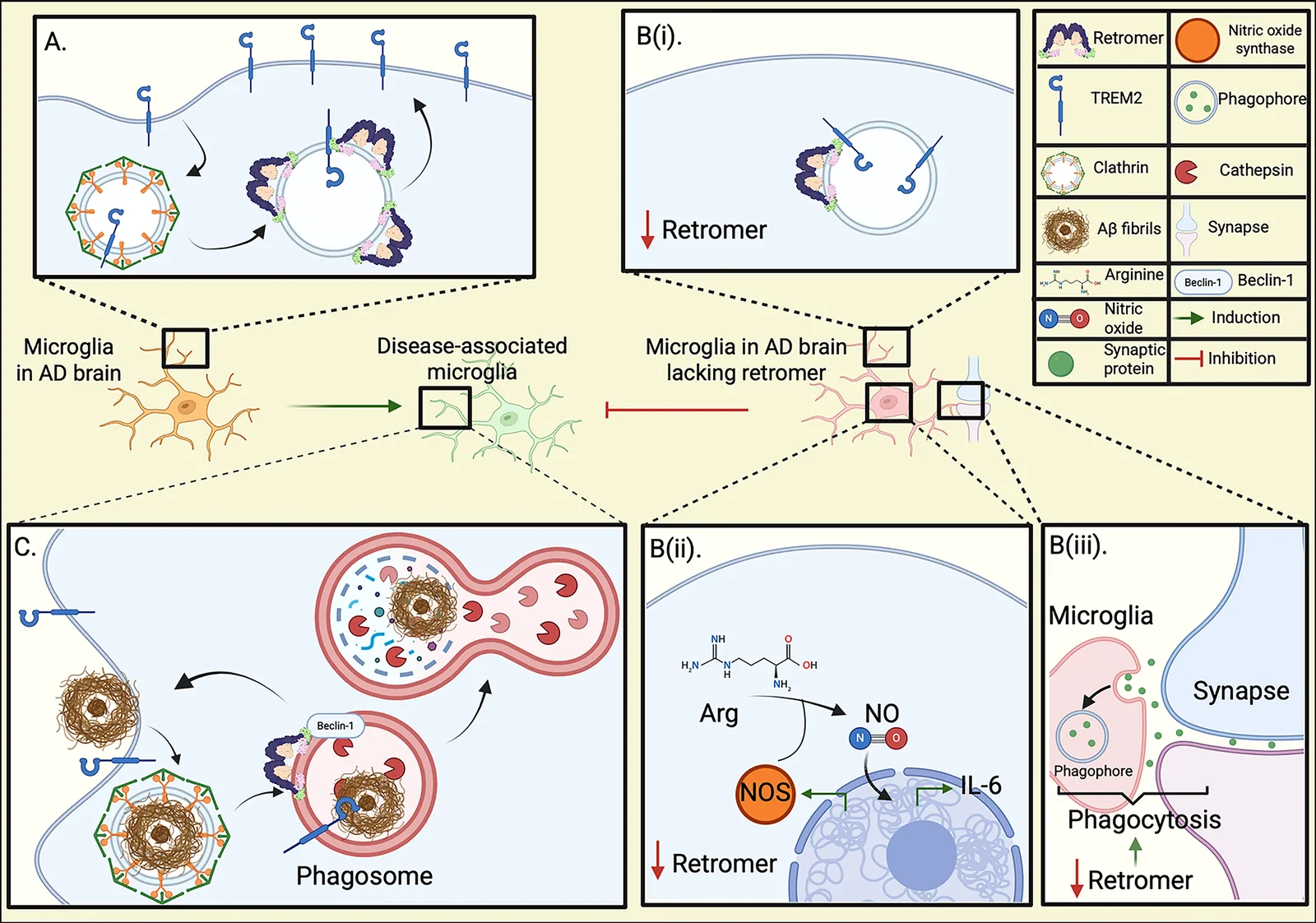
Retromer role in neuroinflammation in Alzheimer’s disease. (**A**) Microglia: TREM2 in the plasma membrane is internalized in a clathrin-dependent manner and recycled back to the plasma membrane through VPS35; (**B**) Microglia lacking the retromer: (i) TREM2 accumulates in the lysosomes, (ii) excessive lipopolysaccharide (LPS)-induced inducible nitric oxide synthase (iNOS) expression and production of the pro-inflammatory cytokine IL-6, (iii) Increased microglial-mediated phagocytosis of hippocampal synaptic proteins. (**C**) Disease-associated microglia (DAM): Microglial-mediated phagocytosis of Aβ via TREM2 in a clathrin-dependent mechanism. VPS35 recruitment to the phagosomal membranes is regulated by Beclin-1. Created in https://BioRender.com

*Alzheimer’s disease:* During AD development, microglia acquire a unique transcriptional and functional signature, evolving into disease-associated microglia (DAM) (Fig. 9). DAM can contain Aβ plaques and protect surrounding neuronal tissue (Fig. 9) [278]. Microglial VPS35 is necessary in DAM development and Aβ uptake [279, 280]. Interestingly, there is a reduction of VPS35 in microglia harvested from AD brains [281]. The retromer enhancer R55 favors a DAM and M2 anti-inflammatory microglia profile in response to the neuroinflammatory state in the AD microenvironment [257]. VPS35 favors Aβ phagocytosis, a DAM and anti-inflammatory profile via recycling TREM2 (triggering receptor expressed on myeloid cells) back to the plasma membrane (Fig. 9) [282]. In fact, microglia require the phagocytic receptor TREM2 for full acquisition of a DAM profile and for phagocytosis [278] and VPS35 deficiency leads to excessive inducible nitric oxide synthase (iNOS) expression and production of the pro-inflammatory cytokine interleukin 6 (IL-6) (Fig. 9) [282]. As mentioned above, microglial VPS35 also regulates microglial Aβ uptake [279]. VPS35 and TREM2 recruitment to the phagosomal membranes is regulated by beclin-1 (Fig. 9) [281].

Apolipoprotein E (APOE) is predominantly expressed by microglia and astrocytes [283] and remains the strongest and most prevalent genetic factor for sporadic AD, impacting more than half of all AD cases [284]. Pathogenically, apoE seeds Aβ plaques in the brain, with the isoform apoE4 driving earlier and more abundant amyloids [284]. Qureshi et al. demonstrated that APOE levels are increased in a genetic mouse model where VPS35 is depleted in hippocampal neurons, and partially normalized by VPS35 overexpression [147]. However, additional studies are necessary to further investigate retromer-mediated APOE regulation.

**Fig. 10:**
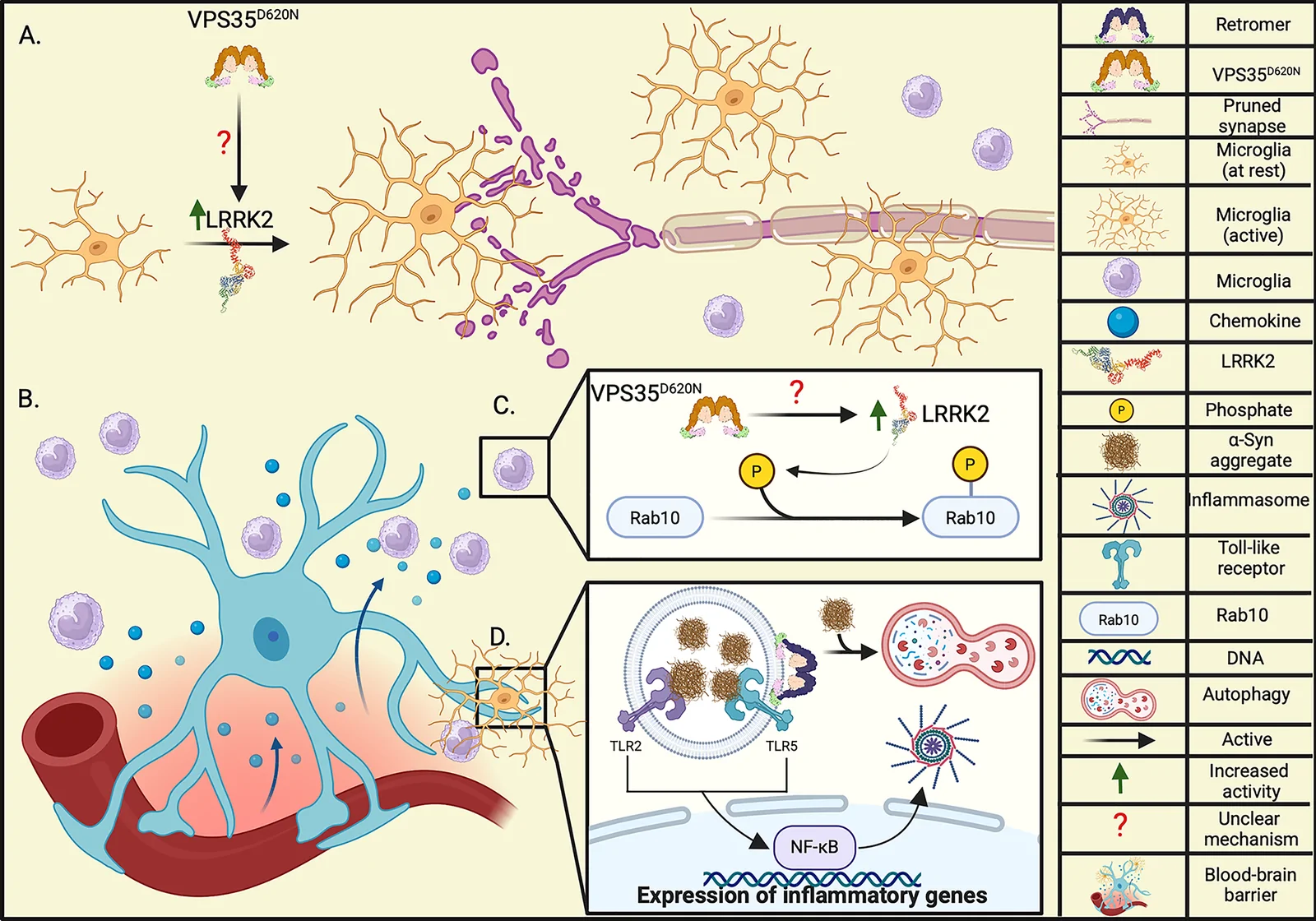
Retromer role in neuroinflammation in Parkinson’s disease. (**A**) Microglia: VPS35^D620N^ mutation, via an unclear mechanism, enhances Leucine-Rich Repeat Kinase (LRRK2) activity, which induces the formation of excessive branches and junctions on microglial cells, leading to excessive microglial-mediated synaptic pruning. (**B**) Monocytes: higher levels of chemokines attracting monocytes are upregulated in the substantia nigra. (**C**) Monocytes: VPS35^D620N^ leads to increased levels of LRRK2-mediated RAB10 phosphorylation via an unclear mechanism. (**D**) Microglia: The retromer modulates the degradation of α-synuclein, which binds Toll-like receptor 2 and 5 (TLR2 and TLR5) and activates the NOD-, LRR- and pyrin domain-containing 3 (NLRP3) inflammasome pathway, leading to microglia activation. Created in https://BioRender.com

*Parkinson’s disease:* Neuroinflammation plays a significant role in PD and ALS neurodegeneration, though there are only early data on the retromer involvement in PD, and limited data in ALS. Oligomeric αS binds toll-like receptor 2 and 5 (TLR2 and TLR5) and activates the NOD-, LRR- and pyrin domain-containing 3 (NLRP3) inflammasome pathway, leading to microglia activation (Fig. 10) [285, 286]. Microglial NLRP3 activation is associated with dopaminergic neuronal loss and PD motor deficits [287]. By modulating different αS degradation pathways [155], the retromer may play an indirect role in the activation of microglia and neurodegeneration. The LRRK2 G2019S mutation induces the formation of excessive branches and junctions on microglial cells, leading to excessive microglial-mediated synaptic pruning and loss of dopaminergic fibers in the dorsal striatum of middle-aged PD mice (Fig. 10) [288]. The LRRK2 G2019S mutation is likely a toxic gain-of-function mutation that augments LRRK2 kinase activity [289]. The VPS35^D620N^ mutation enhances LRRK2 kinase activity even more than the LRRK2 G2019S mutation (Fig. 10) [99]. As such, VPS35^D620N^ may play a similar role in the microglial-mediated dopaminergic synaptic refining, though this has yet to be demonstrated. Monocytes from PD patients carrying the VPS35^D620N^ had increased levels of LRRK2-mediated RAB10 phosphorylation compared to idiopathic PD and healthy controls (Fig. 10) [99]. The role of VPS35-mediated LRRK2 gain of function in relation to immune cell function and neuroinflammation is still unclear.

#### The retromer as a potential therapeutic target

The retromer’s key role in regulating multiple ELS-dependent mechanisms linked to neurodegeneration highlights its potential as a common therapeutic target across different neurodegenerative diseases. In vitro and in vivo studies implementing pharmacological enhancers of the retromer were originally conducted in AD models and revealed rescue from AD phenotypes [154]. These enhancers, TPT-260 (R55), and TPT-172 (R33) are part of the “R-compounds” drug class and were originally introduced by Mecozzi et al. in their seminal 2014 paper [154]. In the VPS35, VPS26, VPS29 heterotrimer, VPS35 is the least stable subunit with a denaturation temperature of 38.5 °C, compared to 56.4 °C for VPS26 and 50 °C for VPS29 [154]. The addition of VPS26 to VPS35 results in a melting temperature of the complex of 53.4 °C, whereas the VPS35–VPS29 and VPS35–VPS29-VPS26 complex denatures closer to the temperature of Vps35 alone (45.5 °C) [154]. After implementing two computational approaches and in silico screening of 46,000 compounds, only 4 docking sites on the VPS35-VPS29 interface were identified for secondary in vitro screening. Of the 24 compounds tested, only one, thiophene-2,5-diylbis(methylene) dicarbamimidothioate dihydrochloride (R55), improved the thermal stability of the core retromer protein complex by 10 °C at the highest concentration tested [154]. R55 is predicted to make interactions with side chains from VPS35 and VPS29 [290]. To identify the R55 binding site, mutagenesis experiments identified the Q538W mutation in VPS35 as preventing R55 from binding [154]. Further studies have shown that the R55 best docking site is a small surface recess surrounded by the Phe541 residue in VPS35, and by Tyr139 and Thr144 residues in VPS29 [103]. R55 doses ranging from 0.5 µM to 50 µM were nontoxic to neurons [154]. Also, VPS35 protein, though not mRNA, levels were increased in neurons manipulated with R55, indicating protein stabilization without an effect on mRNA [154]. In the same study, R33 (or thiophen-2-ylmethyl carbamimidothioate hydrochloride) was investigated and revealed the ability to bind the same pocket of R55, though with reduced potency, as demonstrated by an increase in VPS35 levels that was lower in magnitude compared to the levels observed for R55 [154]. R55 was able to reduce the pathogenic processing of APP and Aβ levels in primary cultures of hippocampal neurons [154]. hiPSC-derived neurons engineered with CRISPR/Cas9 to *APP* KO and APP Swedish mutation cells were treated with R33, showing its effectiveness in reducing Aβ and p-tau levels [291]. Last but not least, the chronic administration of R33 has been shown to improve memory and synaptic integrity, lower Aβ levels and deposition, and decrease tau pathology and neuroinflammation in a transgenic mouse model of AD [152].

An additional retromer-interacting compound, 2a, was identified in 2020 by Muzio et al. [103]. 2a was modeled after R55, replacing the 2,5-disubstituted thiophene scaffold in R55 with a phenyl ring for synthetic versatility purposes and isothioureas with guanylhydrazones, as the latter show good stability and a pKa range closer to neutrality that promotes higher permeability through lipophilic bio-membranes and the blood-brain barrier (BBB) [103]. 2a had significantly higher affinity than R55 for the same R55 binding site (Kd = 1.8 µM and Kd = 128.5 µM, respectively) [103]. In addition, via X-ray diffraction experiments, the crystal structure of compound 2a bound to the VPS35–VPS29 complex has been resolved, identifying two novel binding sites beyond the known R55 binding site, providing structural evidence of target engagement with the retromer complex [165]. VPS35, VPS26b and VPS29 proteins were significantly increased by compound 2a in Neuro2a cells after treatment for 48 h [103]. 2a has been demonstrated to cross the BBB in wild type mice and the SOD1^G93A^ mouse model of ALS, increasing retromer subunit protein levels, CI-MPR and mature form of the lysosomal hydrolase CTSD levels, decreasing misfolded SOD1 protein levels and rescuing the degeneration of MNs and motor defects [103].

More recent data on the effect of 2a in PD have been investigated by our group [155, 292]. Administration of 2a in a PD mouse model increased VPS35 protein levels, retromer cargo proteins, and the mature form of the lysosomal hydrolase CTSD, supporting an enhancement of retromer functional activity [155]. This was associated with decreased oligomeric αS levels and protection against degeneration of SN dopaminergic neurons, highlighting a disease-modifying function [155].

*Challenges in targeting the retromer complex:* One important challenge in targeting the retromer therapeutically is maintaining the stoichiometric balance among its subunits. The retromer functions as a tightly regulated multimeric complex whose subunits exist in equilibrium with each other. This is demonstrated by the ability of the subunits to form higher-order oligomeric arrangements of the retromer at higher subunit concentrations [188]. Additionally, VPS26 depletion also decreased the levels of VPS29 and VPS35 [94, 96, 104]. As such, a stoichiometric imbalance between the retromer subunits could alter the retromer assembly process and function. These observations suggest that selectively increasing a single retromer subunit level or function may not be sufficient and could even disrupt retromer homeostasis. As such, pharmacological strategies should aim to stabilize or enhance the function of the assembled retromer complex rather than disproportionately increasing individual subunits. Small molecules acting as pharmacological chaperones that promote coordinated stabilization of the VPS35–VPS29–VPS26 complex may represent a more balanced and physiologically relevant therapeutic approach. This small-molecule approach may also offer an advantage over retromer genetic overexpression strategies, as it is intended to enhance endogenous retromer function rather than induce supraphysiological increases in individual subunits. 2a increases the levels of all the subunits of the retromer (VPS35, VPS26b and VPS29) [103], and further studies are currently undergoing to investigate the details of this mechanistic effect on the retromer.

Second, given the retromer’s involvement in different cellular pathways, the enhancement of its function may alter cellular homeostasis and trigger unintended downstream effects. In fact, a potential caveat to retromer enhancement implementation in neurodegenerative disease clinical practice is its impact on different cancer hallmarks. In pre-clinical models of gastric carcinomas, VPS35 promotes tumor growth, invasion, and metastasis via integrin-mediated activation of FAK-SRC kinases [293]. In the same model, VPS35 knockdown increased the sensitivity of cancer cells to 5-fluorouracil and cisplatin, highlighting VPS35’s role in chemoresistance and cancer progression [293]. Additional pre-clinical evidence has been demonstrated in other cancer types, among which are hepatocellular and cervical carcinoma, with data revealing VPS35’s role in cancer cell proliferation, invasion, apoptosis and metastasis, genomic instability, and immune evasion of cancer [294–296]. However, it remains unclear whether VPS35 mutations act as oncogenic drivers, with evidence limited to neural progenitor-derived cells in *Drosophila*, highlighting the retromer as a safeguard mechanism against Notch-mediated brain tumor formation [297]. As such, retromer function enhancement via R-compounds or retromer overexpression carries a risk of side effects in patients. This aspect will need to be considered for potential future clinical trials investigating the safety and efficacy of retromer stabilizers in AD, PD and ALS patients. A potential approach could be excluding from the trial any patient with a past medical history of malignancy.

To mitigate these potential risks, ongoing preclinical studies are designed to comprehensively characterize the on-target and off-target effects of retromer-stabilizing small molecules before clinical translation. These studies include mechanistic analyses to define how the pharmacological approach modulates retromer complex activity and the ELS, together with IND-enabling safety assessments, including mutagenicity, genotoxicity, toxicology, and pharmacokinetic profiling, including its plasma and brain concentration, tissue distribution, and BBB penetration. Together, these studies will help define a therapeutic window in which retromer activity can be enhanced while minimizing the risk of perturbing cellular homeostasis, promoting unwanted proliferative pathways, or inducing long-term toxic effects.

## Conclusions

AD, PD, and ALS are neurodegenerative diseases related to each other by similar pathogenetic mechanisms. A central mechanism is the presence of a dysfunctional ELS, which affects four common pathways in AD, PD and ALS. Impaired ELS-regulated proteostasis leads to misfolded proteins, whose accumulation and aggregation likely contribute to neuronal injury and death. Additionally, the ELS is involved in mitochondrial biogenesis and homeostasis dysfunction, with abnormal mitochondrial fragmentation and increased oxidative stress and cellular apoptosis. Deficits in neurotransmitter reuptake and synaptic receptor recycling and clearance are also important. Neuroinflammation is an additional mechanism affected by ELS dysfunction. The retromer is at the cornerstone of each one of these processes, modulating the ELS and rescuing the above-mentioned interconnected processes in diseased neurons and reactive microglia. A relatively novel class of molecular chaperones, enhancers of the retromer, has proven to slow the neurodegenerative phenotype typical of PD and ALS in in vitro and in vivo models [103, 152, 155]. Retromer stabilization’s neuroprotective effect lies in favoring the ELS homeostasis with trafficking of lysosomal hydrolases and cargo receptors, facilitating the degradation of amyloidogenic proteins such as Aβ and αS, as well as misfolded proteins including SOD1 and TDP-43. Beyond its role in protein clearance, retromer stabilization has been shown to dampen neuroinflammation and facilitate neurotransmission. As such, retromer enhancement represents a multifaceted therapeutic strategy leading to neuroprotection across different neurodegenerative disorders. This preclinical evidence highlights the role of retromer enhancers as potential drugs that could step into clinical trials for multiple neurodegenerative conditions.

## Data Availability

No datasets were generated or analysed during the current study.

## Abbreviations

2a: 1,3 bis-phenyl guanylhydrazone
5-HT_4(a)_Rs: 5-hydroxytryptamine 4a receptors
αS: α-Synuclein
β2AR: β2 Adrenergic Receptor
Aβ: Amyloid Beta
AD: Alzheimer’s disease
AICD: Amyloid precursor Intracellular Domain
ALS: Amyotrophic Lateral Sclerosis
AMPAR: α-amino-3-hydroxy-5-methyl-4-isoxazolepropionic acid receptors
APLP2: Amyloid precursor-like protein 2
APOE: Apolipoprotein E
APP: Amyloid Precursor Protein
ATFS-1: Activating Transcription Factor associated with Stress
ATG9A: Autophagy-Related protein 9A
BACE1: β-site Amyloid precursor protein Cleaving Enzyme 1
BAR: Bin, Amphiphysin and Rvs
BBB: Blood-brain barrier
CCC: COMMD/CCDC22/CCDC93
CCDC: Coiled-Coil Domain-Containing protein,
CI-MPR: Cation-independent Mannose Phosphate Receptor
CMA: Chaperone-Mediated Autophagy
CNS: Central Nervous System
COMMD: Copper Metabolism MURR1 Domain-containing protein
CSC: Cargo Selection Complex
CSF: Cerebrospinal Fluid
CTSD: Cathepsin D
CTSL: Cathepsin L
DA: Dopaminergic
DAM: Disease-Associated Microglia
DAT: Dopamine Transporter
DLP1: Dopamine Receptor D1
DRD1: Dopamine Receptor D1
Drp1: Dynamin-related Protein 1
DS: Down syndrome
eIF2α: Eukaryotic translation Initiation Factor 2 subunit 1
EE: Early Endosomes
ELS: Endosomal-Lysosomal System
ESCPE-1: Endosomal sorting complex for promoting exit 1
ESCRT: Endosomal complex required for transport
FAM21: Family with sequence similarity 21
FDA: Food and Drug Administration
Fe: Iron
FERM: 4.1/ezrin/radixin/moesin
GARP: Golgi-associated retrograde protein
GBA: Glucocebrebrosidase
GluA1: Glutamate Receptor Subunit A1
GluR1: Glutamate Receptor 1
GTPase: Guanosine Triphosphatase
hiPSC: human-induced Pluripotent Stem Cells
Hsc70: Heat Shock Cognate Protein 70
HSPA8: Heat Shock Protein Family A Member 8
IL-6: Interleukin-6
KI: Knock-in
KO: Knock-Out
LAMP2A: Lysosomal-Associated Membrane Protein 2A
LC3-II: Light Chain 3-II
LE: Late Endosomes
LRRK2: Leucine-rich Repeat and Fibronectin type-III domain containing protein 2
LTP: Long-Term Potentiation
MA: Macroautophagy
MAPL: Mitochondria Anchored Protein Ligase
MDV: Mitochondrial-Derived Vesicles
MFN: Mitofusin
MN: Motor Neurons
MOM: Mitochondrial Outer Membrane
MOMP: Mitochondrial Outer Membrane Permeabilization
MPTP: 1-methyl-4-phenyl-1,2,3,6-tetrahydropyridine
mtDNA: Mitochondrial DNA
MUL-1: Mitochondrial E3 Ubiquitin Protein Ligase 1
NEK1: NIMA-related kinase 1
NEV: Neuronal Extracellular Vesicles
NLRP3: NOD-, LRR- and pyrin domain-containing 3
NMDAR: N-Methyl-D-Aspartate Receptors
NPxY: Asn-Pro-Xaa-Tyr
PD: Parkinson’s disease
PDZ: PSD95, Dlg1, and zo-1
PI3P: Phosphatidylinositol 3-phosphate
PINK1: PTEN-induced putative kinase 1
PLA2: Phospholipase A2
OPA1: Optic Atrophy 1
OXPHOS: Oxidative Phosphorylation
R33: Thiophen-2-ylmethyl carbamimidothioate hydrochloride
R55: Thiophene-2,5-diylbis(methylene) dicarbamimidothioate dihydrochloride
ROS: Reactive Oxygen Species
sAPP β: Soluble Amyloid Precursor Protein β
SLC7A1: Solute carrier family 7 member 1
SN: Substantia Nigra
SNX: Sorting Nexin
SOD1: Superoxide Dismutase 1
SORL1: Sortilin-related receptor 1
TBC1D5: TBC1 domain family member 5
TDP-43: TAR DNA-binding protein 43
TfR: Transferrin Receptor
TGN: Trans-Golgi network
TLR: Toll-like receptor
TREM2: Triggering Receptor Expressed on Myeloid cells 2
UPR^mt^: Mitochondrial Unfolded Protein Response
UPS: Ubiquitin-Proteasome System
VMAT2: Vesicular Monoamine Transporter 2
VPS: Vacuolar Protein Sorting
VPS35L: VPS35 endosomal protein-sorting factor-like protein
WASH: Wiskott-Aldrich Syndrome protein and scar homologue complex
